# Nuclearity enlargement from [PW_9_O_34_@Ag_51_] to [(PW_9_O_34_)_2_@Ag_72_] and 2D and 3D network formation driven by bipyridines

**DOI:** 10.1038/s41467-022-29370-w

**Published:** 2022-04-04

**Authors:** Zhi Wang, Yan-Jie Zhu, Ying-Zhou Li, Gui-Lin Zhuang, Ke-Peng Song, Zhi-Yong Gao, Jian-Min Dou, Mohamedally Kurmoo, Chen-Ho Tung, Di Sun

**Affiliations:** 1 School of Chemistry and Chemical Engineering, and State Key Laboratory of Crystal Materials, Shandong University, 250100 Ji’nan, People’s Republic of China; 2 Shandong Provincial Key Laboratory of Molecular Engineering, Qilu University of Technology (Shandong Academy of Science), 250353 Ji’nan, People’s Republic of China; 3 College of Chemical Engineering and Materials Science, Zhejiang University of Technology, 310032 Hangzhou, People’s Republic of China; 4 School of Chemistry and Chemical Engineering, Henan Normal University, 453007 Xinxiang, People’s Republic of China; 5 Shandong Provincial Key Laboratory of Chemical Energy Storage and Novel Cell Technology and School of Chemistry and Chemical Engineering, Liaocheng University, 252000 Liaocheng, People’s Republic of China; 6 Université de Strasbourg, Institut de Chimie de Strasbourg, CNRS-UMR 7177, 4 rue Blaise Pascal, 67008 Strasbourg, Cedex France

**Keywords:** Coordination chemistry, Inorganic chemistry

## Abstract

The structural transformations of metal nanoclusters are typically quite complex processes involving the formation and breakage of several bonds, and thus are challenging to study. Herein, we report a case where two lacunary Keggin polyoxometallate templated silver single-pods [PW_9_O_34_@Ag_51_] (SD/Ag51b) fuse to a double-pod [(PW_9_O_34_)_2_@Ag_72_] by reacting with 4,4’-bipyridine (bipy) or 1,4-bis(4-pyridinylmethyl)piperazine (pi-bipy). Their crystal structures reveal the formation of a 2D 4^4^-*sql* layer (SD/Ag72a) with bipy and a 3D *pcu* framework (SD/Ag72c) with pi-bipy. The PW_9_O_34_^9−^ retains its structure during the cluster fusion and cluster-based network formation. Although the two processes, stripping of an Ag-ligands interface followed by fusion, and polymerization, are difficult to envisage, electrospray ionization mass spectrometry provides enough evidences for such a proposal to be made. Through this example, we expect the structural transformation to become a powerful method for synthesizing silver nanoclusters and their infinite networks, and to evolve from trial-and-error to rational.

## Abstract (web summary)

Controlling the structural transformations of metal clusters is challenging. Here, the authors synthesize a silver nanocluster and study the structural changes that take place upon reaction with bipyridine derivatives.

## Introduction

The ubiquity of coordination-dissociation equilibrium of metal clusters in solution with partial retention of their original connectivity within the molecular structure provides a way to change their nuclearities and in some cases, allow for oligomerization to giant homologs^1–6^. Because of the subtle balance of bond energies, for example Ag–S, Ag–N, Ag-O versus Ag···Ag, and geometrical shapes, these reactions have given rise to an unexpected chemistry where coordination rules are not always fully obeyed and is now developing in a preparation process hereafter called structural transformation of metal nanoclusters. This process has been used to create clusters in a more controllable way compared to the classical de novo synthesis of cluster from simple reactants^7–13^. In particular, structural transformation is especially adapted for the assembly of silver clusters because of the versatile coordination geometries of Ag atom (strong coordination bonds) and ubiquitous moderate to weak argentophilic interactions as well as weak hydrogen bonding interactions^14–18^. Such alliance of pre-existing strong and weak interactions in silver clusters favors their subsequent dynamic structure transformation in response to some stimuli while partially retaining the cluster nuclearity, structure and at times its geometry. Currently, the employment of such strategy in the syntheses of silver clusters remains a rare event, although a related one called LEIST (ligand-exchange-induced size/structure transformation) methodology has been developed for reduced Au nanoclusters^19–22^. Related to structural transformation, Mak’s group revealed an induced silver core enlargement from a small Cl@Ag_14_ to a large Cl_6_Ag_8_@Ag_30_ by the reaction with AgClO_4_^23^. Soon afterwards, a discrete Ag_12_ cluster was interconnected into a 3D framework by post-synthetic modification using 4,4’-bipyridine (bipy), while the unchanged silver cluster acts as the secondary building unit in the polymerization process^24^. Zang’s group reported the reversible configuration transformation of the [V^V^_10_V^IV^_2_O_34_]^10−^ core in the Ag_30_ nanocage upon acid/base stimuli^25^. These sporadic reports exemplified the potential of structural transformation of silver clusters by specific stimuli. Motivated by the above advances, our group also successfully realized the synergetic conversion of both core and shell from [Mo_6_O_22_@Ag_44_] to [Mo_8_O_28_@Ag_50_] induced by benzoic acid and deciphered the underlying breakage-growth-reassembly (BGR) transformation mechanism based on a comprehensive characterization using electrospray ionization mass spectrometry (ESI-MS)^26^. This is a very complex and unique structural transformation involving the simultaneous enlargement of both the anionic core template and the outer silver shell.

As an important subclass of polyoxometalates (POMs), lacunary POMs have more O binding sites, higher negative charges and pre-organized region containing asymmetric metal-accessible vacant sites^27–30^, thus should be a kind of powerful anion template to assemble silver clusters. Nonetheless, lacunary POMs templated silver clusters are very seldom which may be caused by the instability of lacunary POMs in solution. Hitherto, only few examples have appeared based on such kind of POMs, [(PW_9_O_34_)_2_@Ag_70_]^31^, [(PW_9_O_34_)_2_@Ag_67_]^32^, [α-SiW_10_O_37_@Ag_41_]^33^, and [SiW_9_O_34_@Ag_51_]^34^. Of note Wang et al. introduced the ionothermal synthesis to address the stability problem of lacunary POMs during the assembly of silver clusters^31^.

In this work, we isolated a pumpkin-like single PW_9_O_34_^9−^ templated Ag_51_ cluster, [(PW_9_O_34_)@Ag_51_(*i*PrS)_25_(CF_3_COO)_17_(DMF)_3_(CH_3_OH)_3_] (**SD/Ag51b**) using a straightforward synthesis method. The coordination of six solvent molecules on the surface of **SD/Ag51b** inspired us to apply structural transformation by employing bridging poly-pyridine ligands such as bipy and 1,4-bis(4-pyridinylmethyl)piperazine (pi-bipy). As expected, a 2D 4^4^-*sql* layer {[(PW_9_O_34_)_2_@Ag_72_S(*i*PrS)_41_(CF_3_COO)_8_(bipy)_5.5_(CH_3_OH)(H_2_O)]·3CF_3_COO}_*n*_ (**SD/Ag72a**) and a 3D *pcu* framework {[(PW_9_O_34_)_2_@Ag_72_S(*i*PrS)_42_(CF_3_COO)_7_(pi-bipy)_4.5_(CH_3_OH)]·3CF_3_COO}_*n*_ (**SD/Ag72c**) containing peanut-like double PW_9_O_34_^9−^ templated 72-nuclei silver cluster as node were isolated. Such structural transformation examples involve the simultaneous increase of the nuclearity of the silver cluster and network formation of different dimensionalities induced by two closely related bipy.

## Results

**Fig. 1:**
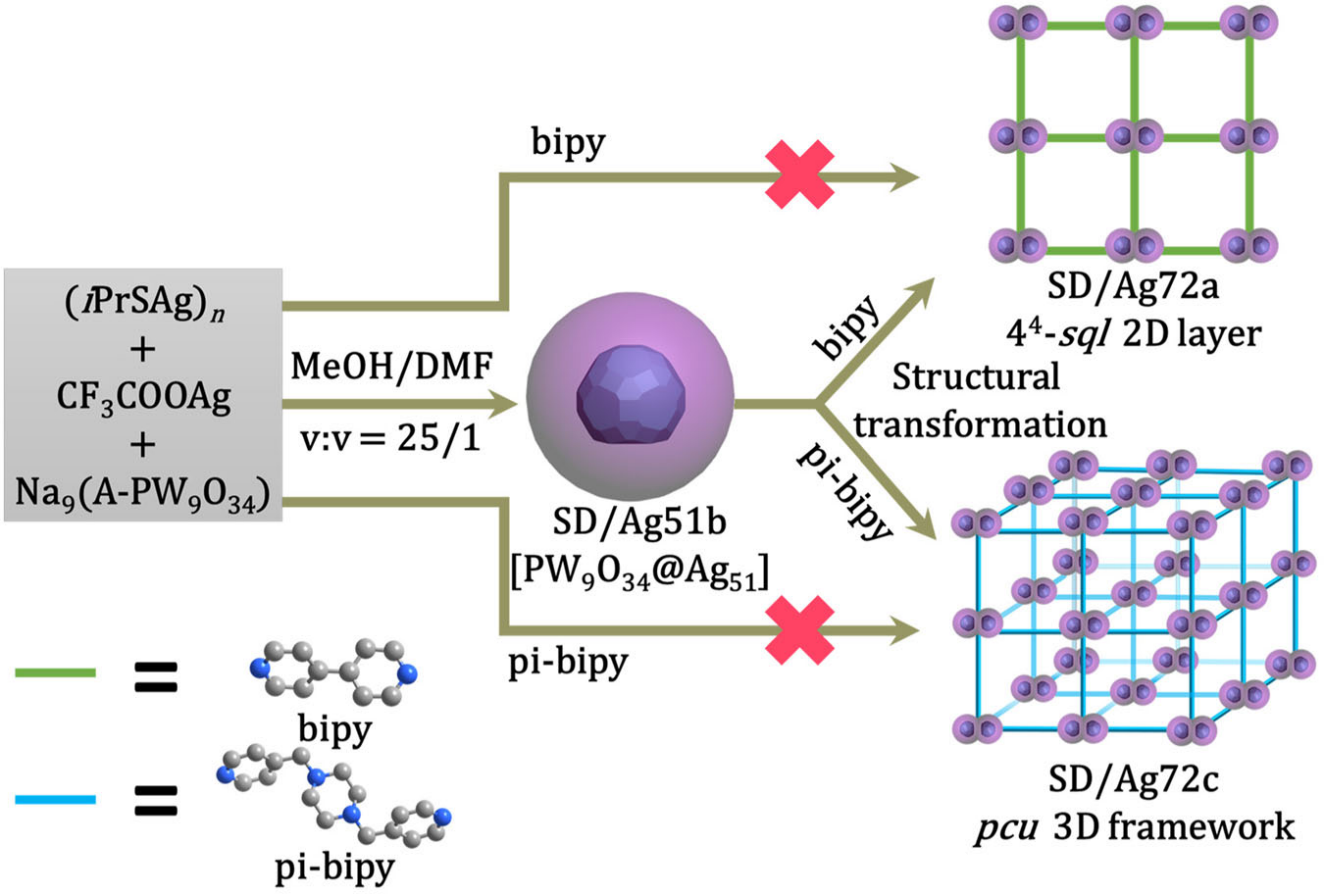
Schematic representation of the structural transformation of **SD/Ag72a** and **SD/Ag72c** from **SD/Ag51b**. Upper: The bipy induced **SD/Ag51b** to form 4^4^-*sql* 2D layer of **SD/Ag72a**. Lower: The pi-bipy induced **SD/Ag51b** to form 3D *pcu* framework of **SD/Ag72c**.

**Synthesis**. Colorless block crystals of **SD/Ag51b** are formed from the reaction of (*i*PrSAg)_*n*_, CF_3_COOAg, and Na_9_(A-PW_9_O_34_)·7H_2_O in CH_3_OH/DMF at 25 °C. Subsequent addition of bipy to the mother liquor of above reaction system yields light yellow needles of **SD/Ag72a** (Fig. 1). Also reaction of bipy with crystals of **SD/Ag51b** dissolved in CH_3_OH leads to **SD/Ag72a** (Fig. 1). But if the bipy is added before the formation of **SD/Ag51b**, **SD/Ag72a** is not obtained. This confirms that the preformed **SD/Ag51b** is the key precursor to **SD/Ag72a** while partially retaining its molecular structure (see below). This is also in line with ESI-MS results (see later). We can then stress that structural transformation is indispensable for generating crystalline silver clusters that are unreachable using traditional one-pot reaction. We also tried to respectively add 1,4-bis(pyrid-4-yl)benzene and pi-bipy to the methanol solution of **SD/Ag51b**, but only latter gave brown-yellow crystals of **SD/Ag72c** (Supplementary Fig. 2). A sequence of characterization techniques such as Fourier transform-infrared spectroscopy (FTIR), UV-Vis spectroscopy, DFT calculations, fluorescence spectroscopy, energy-dispersive X-ray spectroscopy (EDS), single-crystal X-ray diffraction (SCXRD), and powder X-ray diffraction (PXRD), were used in this system (Supplementary Figs. 20–38).

### X-ray structures of SD/Ag51b and SD/Ag72a

**Fig. 2:**
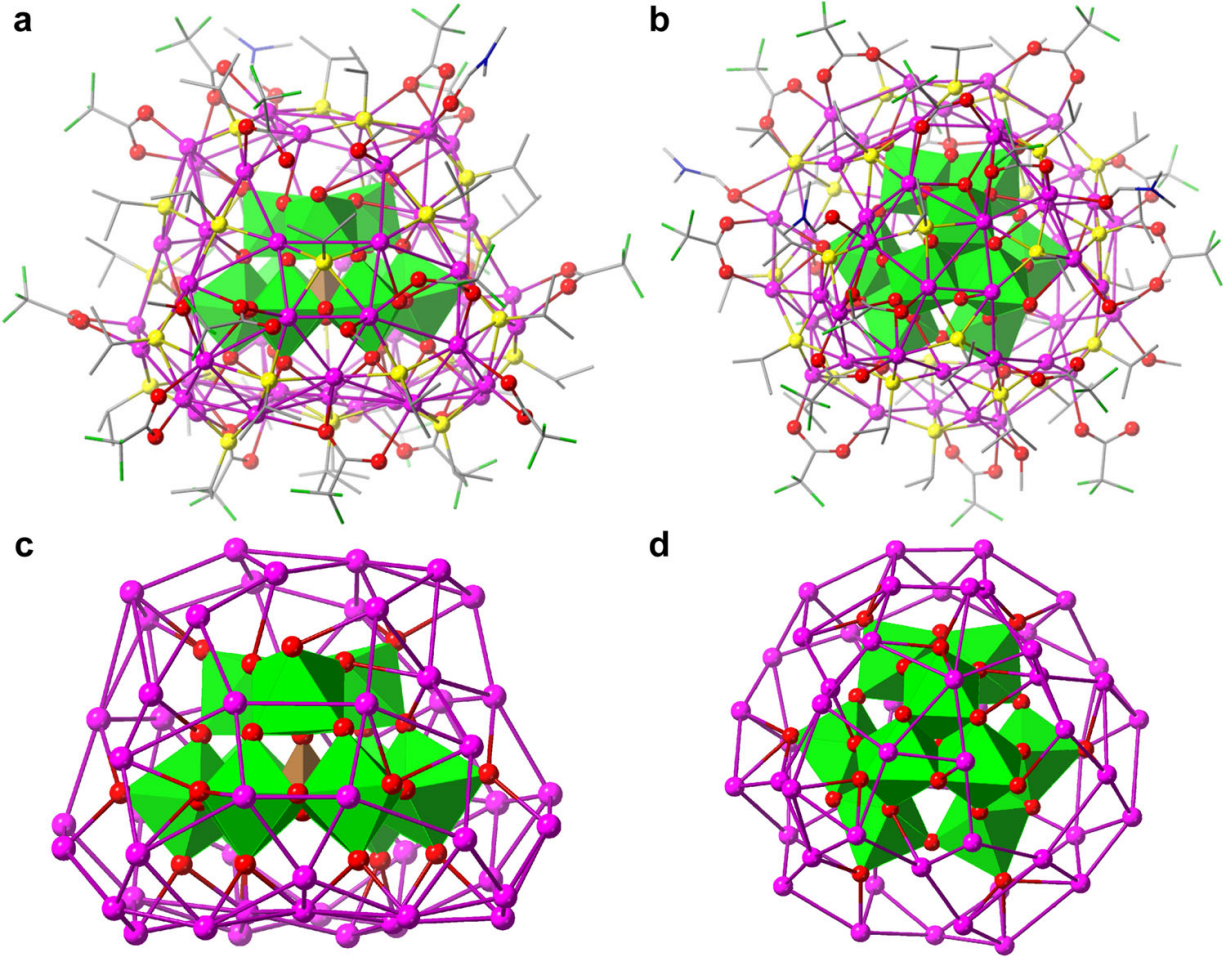
Crystal structure of SD/Ag51b. **a**, **c** Side and (**b**, **d**) top views including (top) and excluding (bottom) the ligands. Color labels: purple, Ag; blue, N; yellow, S; gray, C; red, O; green, F. All H atoms are omitted. PW_9_O_34_^9−^ is shown in polyhedral mode with PO_4_ and WO_6_ colored as brown and green.

SCXRD of **SD/Ag51b** (triclinic, space group *P*-1) found a complete cluster as the asymmetric unit. It can be described as a multishell ball consisting of {PO_4_}@{WO_6_}_9_@Ag_51_@ligands with a cross-section of 1.1 × 1.4 nm^2^ excluding the outer ligands. Based on the molecular building units and for simplicity, we describe it as a single-pod with a core (POM, PW_9_O_34_^9−^) wrapped by the shell (Ag-ligands). The PW_9_O_34_^9−^ core retains its structure as in Na_9_(A-PW_9_O_34_)·7H_2_O (Figs. 2a, b). This lacunary Keggin POM acts as template for the pumpkin-like Ag_51_ shell (Figs. 2c, d) through Ag-O interactions (2.30–2.79 Å) involving coordination to a total of 37 Ag atoms (Supplementary Fig. 3). The silver coordination numbers, not including Ag···Ag contact, vary from 3 (T or Y shape), 4 (seesaw or tetrahedron) to 5 (square-pyramid) (Supplementary Fig. 4). There are 25 *i*PrS^−^, 17 CF_3_COO^−^, 3 terminal DMF and 3 CH_3_OH bonded to and protecting the silver shell. All *i*PrS^−^ show a μ_4_ mode through the sulfur except for two μ_3_-*i*PrS^−^ (Ag-S = 2.32–2.88 Å), and the 17 CF_3_COO^−^ show four different coordination modes (μ_1_-κ^1^:κ^0^, μ_2_-κ^1^:κ^1^, μ_3_-κ^1^:κ^2^, and μ_4_-κ^1^:κ^3^; Ag-O = 2.19–2.78 Å). The silver shell is further consolidated by numerous argentophilic interactions (Ag···Ag = 2.84–3.41 Å). It is an example where the PW_9_O_34_^9−^ is individually wrapped by a silver shell, in contrast to being in pairs in Ag_70_^31^ and Ag_67_ clusters^32^.

**Fig. 3:**
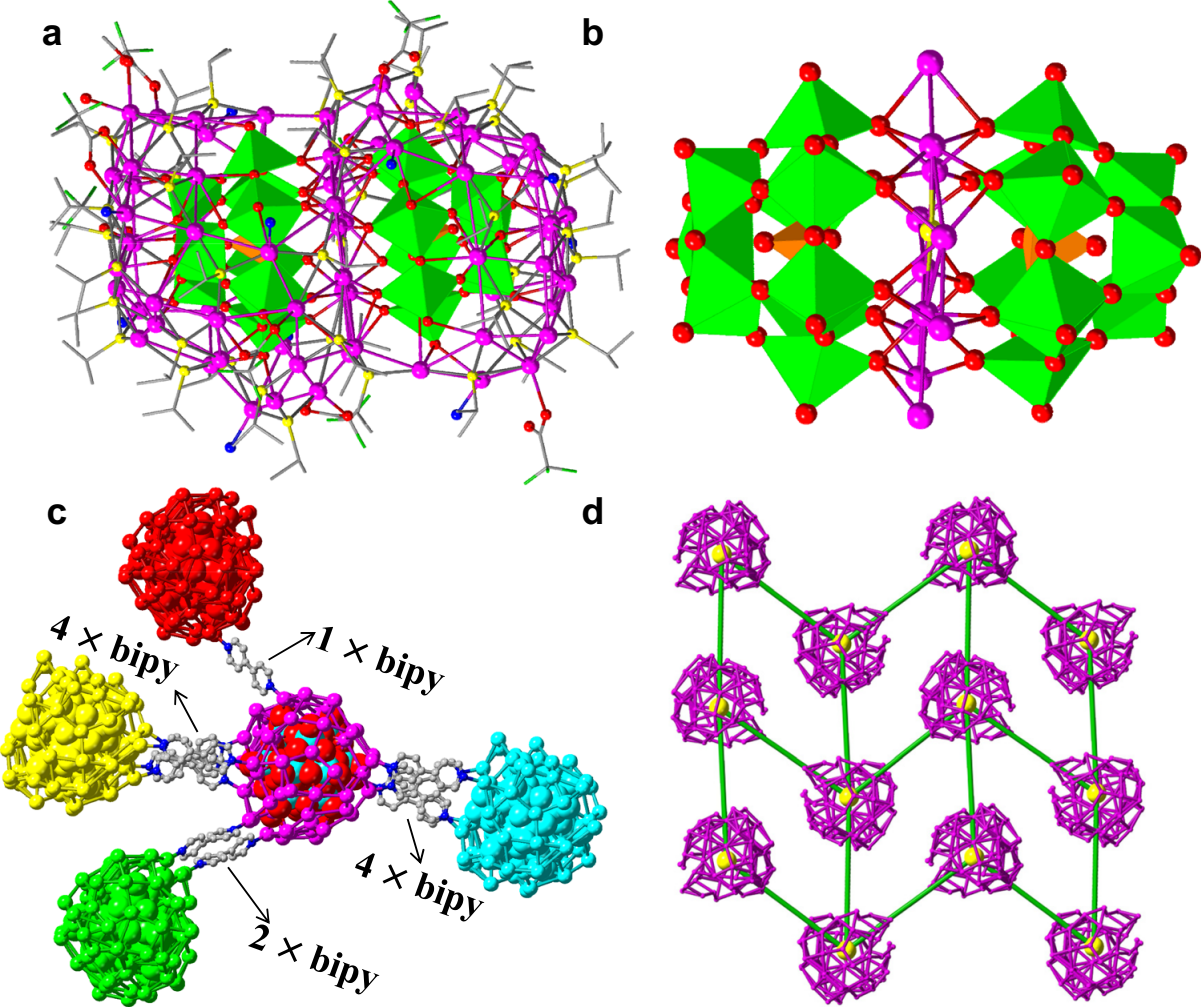
Crystal structure of SD/Ag72a. **a** [(PW_9_O_34_)_2_@Ag_72_] cluster with the ligands and (**b**) highlighting the sandwiched S^2−^-centered silver plane by two PW_9_O_34_^9−^. Color labels: same as Fig. 1. Only N atoms of coordinated bipy are shown for clarity. **c** The connections between [(PW_9_O_34_)_2_@Ag_72_] clusters. PW_9_O_34_^9−^ is shown in space-filling mode. **d** The simplified 2D 4^4^-*sql* network with [(PW_9_O_34_)_2_@Ag_72_] as node (yellow balls) and bipy as linker (green sticks).

The presence of six coordinated solvent molecules on the surface of Ag_51_ shell (Supplementary Fig. 5) prompted us to explore the possibility of using it as a secondary building unit in extending to polymeric structures. We therefore used bipy as a ditopic connector and successfully isolated a 4^4^-*sql* network (**SD/Ag72a**) but with an unexpected modification to the pristine **SD/Ag51b** cluster where it has gone from a single-pod to a double-pod one. SCXRD of **SD/Ag72a** (monoclinic *P*2_1_/*n*) reveals a double-pod cluster with face-to-face POMs centered by a S^2−^ ion as template. The molecular cross-sectional dimension is 2.1 × 1.0 nm^2^ excluding the ligands. The asymmetric unit has a complete peanut-like 72-nuclei silver cluster, which is protected by 41 *i*PrS^−^ (μ_3_ and μ_4_; Ag–S = 2.29–2.93 Å), 8 CF_3_COO^−^ (μ_1_-κ^1^:κ^0^ and μ_2_-κ^1^:κ^1^; Ag–O = 2.21–2.71 Å), 11 bipy (Ag–N = 2.26–2.38 Å) as well as coordinated CH_3_OH and H_2_O (Fig. 3a). The coordination numbers of silve atoms are now extended to 2 (linear), 3 (T or Y shape), 4 (seesaw or tetrahedron), 5 (square-pyramid) and 6 (octahedron) (Supplementary Fig. 6). The Ag_72_ cluster comprises two hemispheres of Ag_29_ and Ag_31_ sandwiching an equatorial S^2−^-centered Ag_12_ plane (Fig. 3b and Supplementary Fig. 7). In each silver hemisphere, a PW_9_O_34_^9−^ is encapsulated as template which supports the outer shell and connects the equatorial S@Ag_12_ plane using the lacunary face of the PW_9_O_34_^9−^. There are 52 of 72 Ag coordinated to two PW_9_O_34_^9−^ through Ag-O bonds (2.29–2.79 Å). The structure of PW_9_O_34_^9−^ in **SD/Ag72a** is the same as that in **SD/Ag51b**, indicating the robustness of PW_9_O_34_^9−^ even after experiencing transformation reaction. Importantly, each [(PW_9_O_34_)_2_@Ag_72_] cluster is connected to four neighbors through a total of 11 bridging bipy (Fig. 3c) to form a 2D 4^4^-*sql* network (Fig. 3d and Supplementary Fig. 8). The two take-away information from these observations are (a) the structural transformation of a low nuclearity silver cluster to a higher one while retaining its templating POM intact but with a slightly modified Ag shell, and (b) the connection of these clusters, as secondary building units, by the ditopic bipy to a coordination polymer.

Of note **SD/Ag72a**, [(PW_9_O_34_)_2_@Ag_72_], is the largest PW_9_O_34_-templated silver cluster so far, followed by [(PW_9_O_34_)_2_@Ag_70_]^31^ and [(PW_9_O_34_)_2_@Ag_67_]^32^. Their similarities and differences give interesting information in the field of giant silver clusters. Their overall geometric shapes which are dominated by that of the templated POM are almost the same and they are all double-pods with two cores (POM, PW_9_O_34_^9−^) wrapped by the shell (Ag-ligands). The major differences appear in the Ag-ligand shells (Supplementary Fig. 9). To simplify the discussion we define two parts: one is separator between the two pods and the other is the outer hemisphere. For [(PW_9_O_34_)_2_@Ag_70_] the separator is an almost flat plane of Ag_10_ and the hemispheres are equal consisting of Ag_30_. For [(PW_9_O_34_)_2_@Ag_67_] the separator is Ag_12_ and the hemispheres are unequal, Ag_28_ and Ag_27_. **SD/Ag72a** has a centrally placed S^2−^ within an Ag_12_ as separator. In spite of the structurally undisturbed anion template, the differences in the overall silver skeletons are likely driven by the capping ligands with different electronic and steric effects, such as *i*PrS^−^, CF_3_COO^−^, bipy for **SD/Ag72a**, *t*BuC ≡ C^−^ for [(PW_9_O_34_)_2_@Ag_70_] and *p*-F-PhS^−^, CF_3_COO^−^ for [(PW_9_O_34_)_2_@Ag_67_]. Other factors include the divergence of the bipy and supramolecular interactions.

### Structure transformation from SD/Ag51b to SD/Ag72a

**Fig. 4:**
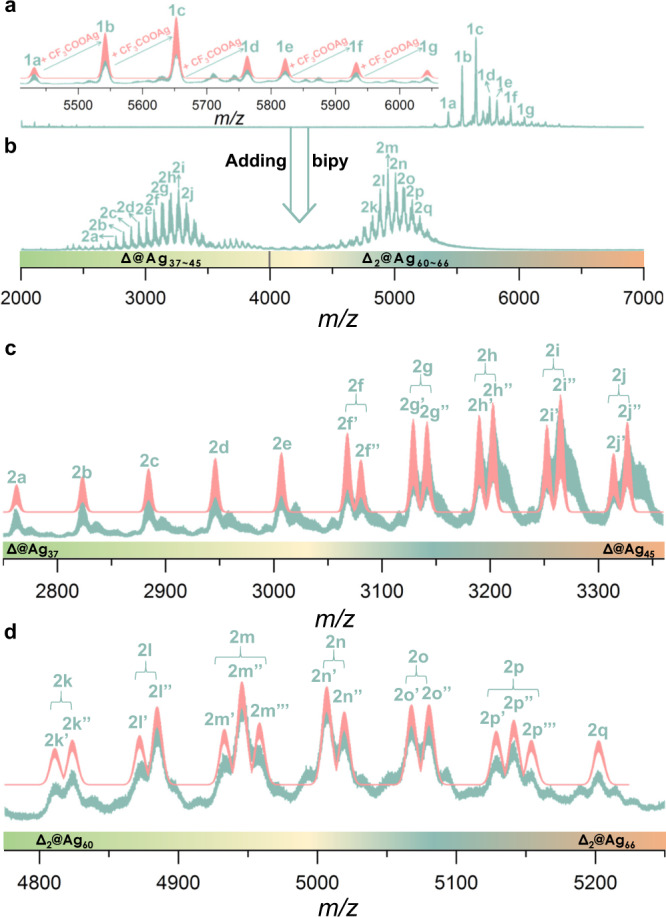
Electrospray ionization mass spectrometry (ESI-MS) of SD/Ag51b before and after adding bipy. Positive-ion ESI-MS of **SD/Ag51b** dissolved in CH_3_OH before (**a**) and after adding bipy (**b**); Δ = PW_9_O_34_^9−^. The charge states for all labeled species in (**a**) and (**b**) are +2 and +3, respectively. Inset: The expanded experimental (green line) and simulated (red line) isotope-distribution patterns of **1a**–**1g**. The expanded experimental (green line) and simulated (red line) isotope-distribution patterns of **2a**–**2j** (**c**) and **2k**–**2q** (**d**).

The structural transformation procedure indicates the formation of **SD/Ag72a** is only possible after the formation of **SD/Ag51b**; that is introduction of bipy to the mother liquor or following isolation and subsequent reaction (Supplementary Fig. 1). Furthermore, using bipy as a reactant before the formation of **SD/Ag51b** gave negative results. Thus, regardless of the route taken, it is important to verify the stability of **SD/Ag51b** in CH_3_OH, which guarantees the transformation reaction genuinely started from Ag_51_ cluster rather than from some fragmented silver species in solution. We performed an ESI-MS study since it has been demonstrated to be a powerful tool to investigate stability and the assembly process of coordination compounds^35–42^. The positive-ion mode ESI-MS of **SD/Ag51b** dissolved in CH_3_OH (Fig. 4a) presents seven key doubly-charged peaks in the *m/z* range 2000–7000. The strongest peak (**1c**: *m/z* = 5652.40) corresponds to a complete cluster [(PW_9_O_34_)@Ag_51_(*i*PrS)_25_(CF_3_COO)_15_]^2+^ (Calcd. *m/z* = 5652.48), which is formed by losing 2 CF_3_COO^−^ and solvent molecules. All other peaks were assigned with precise formulae (Supplementary Table 1) and matched well with their simulated isotopic distributions (Inset of Fig. 4a). The Δ*m/z* for the pairs **1a**–**1b**, **1b**–**1c**, **1c**–**1d**, **1e**–**1f**, **1f**–**1g** are *ca*. 110, which equals the mass of CF_3_COOAg divided by charge state (+2), and indicates the progressive gain of CF_3_COOAg. The peak (**1e**; *m/z* = 5821.55) can be seen as a solvate addendum of **1d** and identified to be [(PW_9_O_34_)@Ag_52_(*i*PrS)_25_(CF_3_COO)_16_(H_2_O)_3_(CH_3_OH)_2_]^2+^ (Calcd. *m/z* = 5821.47). There is no bigger fragment, ruling out the possibility of solvent-induced formation of **SD/Ag72a**. The ESI-MS of the reaction mother solutions during the synthesis of **SD/Ag51b** shows the same peaks **1a**, **1b**, **1c** and **1d** but of low intensities (Supplementary Fig. 10), which suggest that formation and fragmentation follow the same fashion to mentioned above. Furthermore, **SD/Ag51b** can be recrystallized from CH_3_OH (Supplementary Fig. 11). These results confirm: (i) **SD/Ag51b** retains the integrity of its metallic skeleton in CH_3_OH, (ii) **SD/Ag51b** exists in slightly different forms in solution through surface coordination/disassociation of the labile ligands, (iii) **SD/Ag72a** can only be obtained from **SD/Ag51b** using a structural transformation way.

**Fig. 5:**
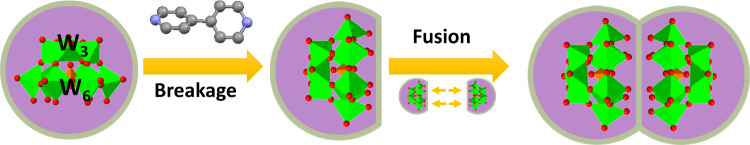
Proposed breakage-fusion conversion mechanism from **SD/Ag51b** to **SD/Ag72a**. The green shell represents silver shell.

The most intriguing question that arises from the structural transformation is how **SD/Ag51b** transforms to **SD/Ag72a** after adding bipy to the solution? ESI-MS provide such insights into the evolution of solution species. Introduction of bipy into a methanol solution of **SD/Ag51b** initiated a synchronous cluster destruction and growth, which are revealed by two separated groups of +3 charged peaks before and after *m/z* = 4000, respectively (Fig. 4b). Below *m/z* = 4000, a total of ten peaks originating from 15 different species were identified (Fig. 4c). All contain a single PW_9_O_34_^9−^ template enwrapped by a shell smaller than Ag_51_, (PW_9_O_34_)@Ag_37–45_. Among them, the most dominant species of **2i”** can be assigned to [(PW_9_O_34_)@Ag_44_(*i*PrS)_22_(CF_3_COO)_10_(H_2_O)_2_]^3+^. Each grouped peak in **2f**–**2j** contains two overlapped species with one *i*PrS^−^ in former replaced by one CF_3_COO^−^ in latter. Above *m/z* = 4000, another 15 species overlapping in seven peaks were assigned to double PW_9_O_34_^9−^ templated clusters of nuclearity higher than 51, (PW_9_O_34_)_2_@Ag_60–66_ (Fig. 4d). They are very likely intermediates towards Ag_72_. The main species in this group are (PW_9_O_34_)_2_@Ag_62_ having different *i*PrS^−^:CF_3_COO^−^ ratios (**2** **m’**, **2** **m”**, and **2** **m”’**). All thirty species were precisely assigned based on simulated and observed isotopic patterns (Supplementary Table 2). The result indicates the lability of the ligands allows for their rapid exchange which promotes the silver shell of **SD/Ag51b** to be dynamic to the formation of the intermediates toward the final **SD/Ag72a**. In comparison with the plenary POMs, the PW_9_O_34_^9−^ possesses more bare-oxygen vacancies and a higher negative charge, therefore, the W_6_ face at the base of the pumpkin-like Ag_51_ cluster may be more active and have higher affinity with the silver atoms than the W_3_ face. Correspondingly, the silver atoms attached to the W_6_ face are also endowed with high reactivity. These highly active regions may be preferentially chosen the way of face-fusion to form a stable entity. Combined with the above observations, we proposed a bipy-induced breakage-fusion conversion mechanism for this system (Fig. 5). The successful isolation of **SD/Ag72a** is caused by the disappearance of the smaller **SD/Ag51b** and this kind of fusion represents a simple model to explain usual coalescence of surfactant-protected nanoparticles from discrete nanocrystals or nanoclusters. The structural transformation was further elucidated by the HAADF-STEM, where the size of the nanoparticles increased from approximately 1.3 to 2.1 nm and some aggregation behavior of them were observed after adding bipy to the methanol solution of **SD/Ag51b** (Supplementary Fig. 12).

The ^31^P NMR was also performed to verify this question (Supplementary Fig. 13). A single resonance peak at δ = −10.24 ppm corresponding to PO_4_^3−^ was observed for **SD/Ag51b** dissolved in methanol, corroborating with one crystallographically independent PW_9_O_34_^9−^. However, the resonance signal of PO_4_^3−^ completely disappear in the −350 to 250 ppm range after adding bipy with the color changing from colorless to yellow. When ejecting the NMR tube from instrument, we surprisingly found abundance of tiny crystals precipitated from the solution, which were confirmed to be **SD/Ag72a** by unit cell checking on a single crystal X-ray diffractometer (Supplementary Fig. 14). The formation of insoluble **SD/Ag72a** decreased the concentration of PO_4_^3−^-contained species in solution, as a result, no signal can be detected after conversion reaction. All these results unambiguously evidenced the occurrence of cluster conversion reaction by addition of bipy.

### Universality of bipy-induced transformation

**Fig. 6:**
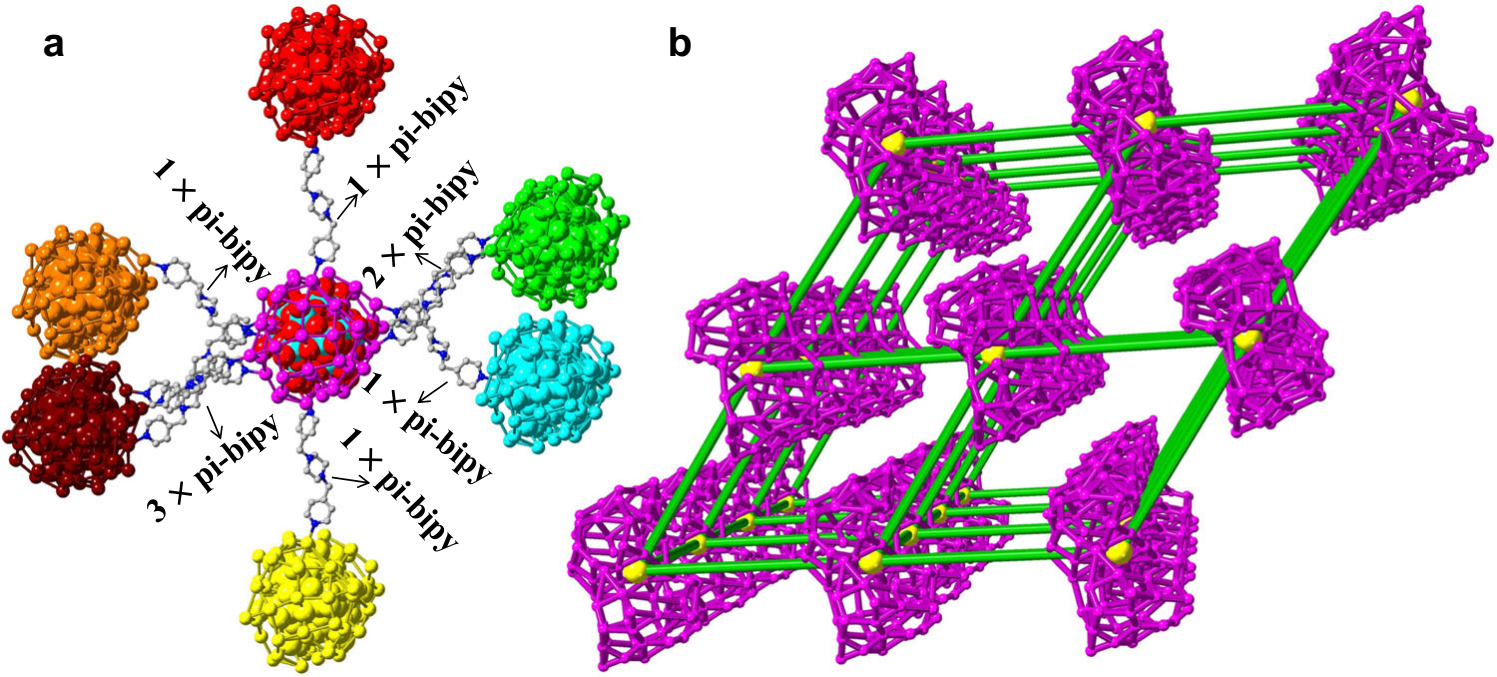
Crystal structure of SD/Ag72c. **a** The connections between [(PW_9_O_34_)_2_@Ag_72_] clusters in **SD/Ag72c**. PW_9_O_34_^9−^ is shown in space-filling mode. **b** The simplified 3D *pcu* framework with [(PW_9_O_34_)_2_@Ag_72_] as node (yellow balls) and pi-bipy as linker (green sticks).

Followed by above successful case, we further evidenced the universality of structural transformation strategy and isolated **SD/Ag72c** as a *pcu* framework by adding pi-bipy to the methanol solution of **SD/Ag51b**. SCXRD analysis shows that the asymmetric unit in **SD/Ag72c** is very similar to that in **SD/Ag72a** (Supplementary Fig. 15). Differently, each [(PW_9_O_34_)_2_@Ag_72_] cluster in **SD/Ag72c** is connected to six neighbors through a total of 9 bridging pi-bipy ligands (Fig. 6a) to form a 3D *pcu* framework (Fig. 6b).

### UV–Vis absorption spectra and luminescence properties

The solid-state UV/Vis spectra of **SD/Ag51b** and **SD/Ag72a** at room temperature are shown in Supplementary Fig. 16. Both exhibit single intense absorption centered at 341 and 352 nm, respectively. Given their structural relevance, the structurally simplified model **SD/Ag51b** was used as a representative to analyze their electronic transitions by means of TD-DFT calculations (See details in Supplementary Information). The absorptions above 400 nm can be ascribed to the ligand and metal charge transfer to the inner core (herein named after L[C]CT and M[C]CT, respectively), i.e. transitions from the surface ligands (mainly 3*p* orbitals of S atoms in the thiol ligands and 2*p* orbitals of O atoms in the carboxylic ligands) and Ag atoms (mainly 4*d* orbitals) to the W = O π* orbitals (formed by 5*d* orbitals of W atoms and 2*p* orbitals of O atoms) in the inner PW_9_O_34_ core; such type of transitions were also previously found in some molybdate-templated Ag clusters^26^. Another two types of transitions, such as those from Ag 4*d* to its 5 *s*/*p* orbitals (MMCT, metal to metal charge transfer) and those from surface S/O *p* orbitals to Ag 5 *s*/*p* orbitals (LMCT, ligand to metal charge transfer), also becomes apparent in the higher-energy region, as exemplified by the typical transitions of HOMO-4/HOMO-6→LUMO + 9 and HOMO-13→LUMO + 3 (Supplementary Table 3 and Data 1). The calculated UV spectrum shows an absorption maximum at 338 nm, which is in fair agreement with the experimental value (300 nm) of **SD/Ag51b** (Supplementary Fig. 17).

**Fig. 7:**
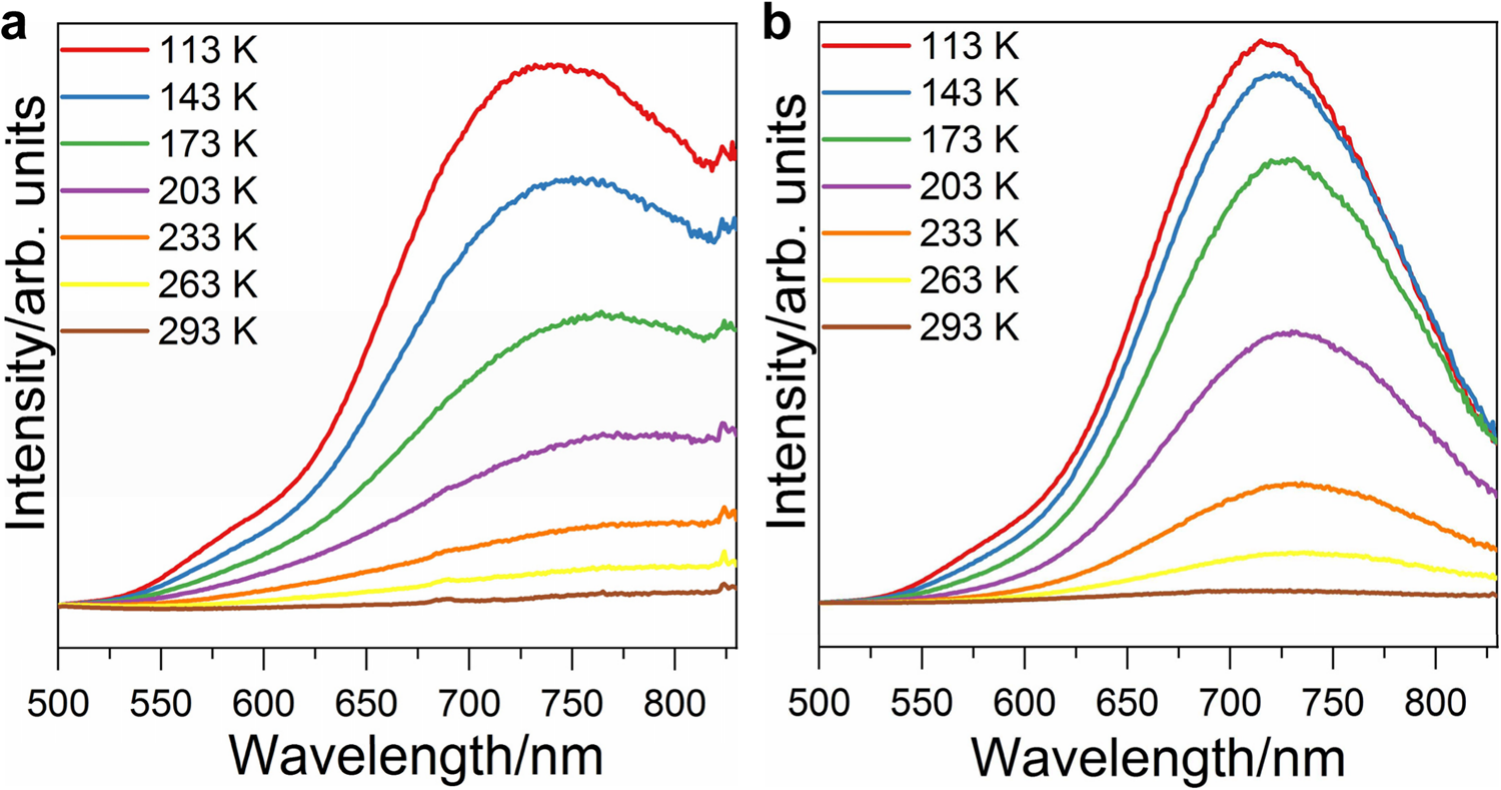
Luminescence spectra of SD/Ag51b and SD/Ag72a. Variable-temperature emission spectra for **SD/Ag51b** (**a**) and **SD/Ag72a** (**b**).

The experimental variable-temperature (113–293 K) solid state emission spectra of **SD/Ag51b** and **SD/Ag72a** under the excitation of 468 nm are shown in Fig. 7. Both emit luminescence in the near-infrared (NIR) region when the temperature is cooling to 263 K with emission peaking at 787 and 737 nm, respectively. Their emission peaks gradually blue-shift to 740 nm for **SD/Ag51b** and 716 nm for **SD/Ag72a** upon cooling to 113 K along with the increase of intensity. The quantum yields of **SD/Ag51b** and **SD/Ag72a** at 113 K were measured to be 1.26% and 2.17%, respectively. The average lifetimes of the emissions are also determined to be on the microsecond scale (τ = 219.4 μs for **SD/Ag51b** and τ = 208.1 μs for **SD/Ag72a**), respectively (Supplementary Fig. 18), indicating their phosphorescence character^43^. Supplementary Fig. 19 shows good linearity correlation between maximum emission intensity (*I*_max_) and temperature (*T*) in the ranges of 113–233 K (**SD/Ag51b**) and 113–293 K (**SD/Ag72a**), which indicate both of them may be promising materials for low-temperature molecular luminescent thermometer. Due to the restriction of theoretical method and computational cost, it is nearly impossible to utilize TD-DFT or other ab-initio method to identify emission mechanism (e.g. electronic transitions, quantum yield) for such larger clusters of **SD/Ag51b** and **SD/Ag72a** with more than 300 non-H atoms. Fortunately, studying electronic transitions of UV absorption is helpful to understand emission property. Based on the calculated absorption results, the emissions of **SD/Ag51b** and **SD/Ag72a** should be associated with the above three types of charge transfers, though the exact excitation/relaxation pathway is still difficult to be determined. According to the previous findings, however, these NIR emissions is more likely to be attributed to the ligand-to-metal charge transfer (LMCT) from S/O *p* to Ag 5 *s*/*p* orbitals perturbed by Ag···Ag interaction^44, 45^. The lowest-energy electronic excitation of the model **SD/Ag51b** in gaseous phase, relevant to its fluorescent emission, is calculated to occur at 474 nm and mainly contributed by the transition from HOMO to LUMO. However, the absorption spectrum of **SD/Ag51b** both experimentally and theoretically in solid state shows a longer-wavelength tail beyond 500 nm. It is demonstrated that a lower excitation energy is sufficient to trigger the transition of S_0_ → S_1_ in solid state and then gives rise to a longer-wavelength emission by further relaxation of S_1_. In this regard, the NIR emissions in **SD/Ag51b** and **SD/Ag72a** are reasonable and can be partially rationalized by the intermolecular interactions in solid state, which often leads to significant red-shift for the emission by forming possible excimers^46–48^.

## Discussion

The key message of this work is the successful application of the structural transformation from an isolated PW_9_O_34_^9−^ templated single-pod Ag_51_ cluster to a double-pod Ag_72_ cluster by the stimulus of bipy while partially retaining the original bonding, shape and the template. Importantly, the linkers bipy and pi-bipy connect the generated Ag_72_ into a 2D corrugated polymeric sheet and a 3D framework, respectively. Thus, the Ag_51_ cluster can be viewed as an intermediate en-route to larger progeny. The structural transformation involves the increase of the nuclearity of silver cluster by trapping more POM templates and the extension of the discrete cluster to infinite 2D and 3D coordination networks by installing the exotic linkers. A breakage-fusion conversion mechanism was also established via reaction monitoring by ESI-MS. This work not only establishes a well-controlled method for synthesizing larger silver nanoclusters but also deepens our understanding on the structural variability and chemical reactivity of this class of silver clusters.

## Methods

### Synthesis of (*i*PrSAg)_*n*_

(*i*PrSAg)_*n*_ was prepared by the following reported procedure^6^. The solution of AgNO_3_ (30 mmol, 5.1 g) in 75 mL acetonitrile was mixed with 100 mL ethanol containing *i*PrSH (30 mmol, 2.8 mL) and 5 mL Et_3_N under stirring for 3 h in the dark at room temperature, then the yellow powder of (*i*PrSAg)_*n*_ was isolated by filtration and washed with ethanol and ether, then dried in the ambient environment (yield: 97 %).

### Synthesis of Na_9_(A-PW_9_O_34_)·7H_2_O

Na_9_(A-PW_9_O_34_)·7H_2_O was prepared by the following reported procedure^49^. Na_2_WO_4_·2H_2_O (0.36 mol, 120 g) was dissolved in l50 g of water and phosphoric acid (85%) was added dropwise with stirring (0.06 mol, 4.0 mL). After addition of the acid is complete, the measured pH was 8.9 to 9.0. Glacial acetic acid (0.40 mol, 22.5 mL) was added dropwise with vigorous stirring. Large quantities of white precipitate form during the addition. The final pH of the solution was 7.5 ± 0.3 and the precipitate is collected after the solution stirring for 1 h then dried in air (yield: 86%).

### Synthesis of SD/Ag51b

(*i*PrSAg)_*n*_ (0.05 mmol, 9.2 mg) and Na_9_(A-PW_9_O_34_)·7H_2_O (0.003 mmol, 7.7 mg) were dissolved in MeOH:DMF (5 mL, v:v = 25:1). After stirring the solution for 1 h at 700 r/min, CF_3_COOAg (0.18 mmol, 39.8 mg) was added and the reaction continued for 3 h under the same condition. The colorless solution was filtrated and the filtrate was evaporated slowly in darkness at room temperature. Colorless block crystals were obtained in 63% yield after 1 week. Selected IR peaks (cm^−1^): 3687 (w), 2959 (w), 1644 (m), 1516 (w), 1449 (w), 1371 (m), 1242 (w), 1195 (m), 1135 (m), 1045 (m), 1033 (m), 884 (w), 830 (m), 797 (m), 712 (s), 599 (m), 498 (w).

### Synthesis of SD/Ag72a

To the mother solution of **SD/Ag51b**, 4,4′-bipyridine (0.16 mmol, 25 mg) was added and stirred for 3 h. The colorless solution was filtrated and the filtrate was evaporated slowly in darkness at room temperature. Faint yellow crystals were obtained in 20% yield after 1 week. Selected IR peaks (cm^−1^): 2950 (w), 1952 (m), 1237 (w), 1199 (m), 1127 (m), 1051 (m), 996 (w), 902 (w), 776 (s), 717 (s), 691 (s), 610 (m), 506 (m).

### Synthesis of SD/Ag72c

To the methanol solution of **SD/Ag51b**, 1,4-bis(4-pyridinylmethyl)piperazine (0.04 mmol, 10 mg) was added and stored in darkness at room temperature. Brown yellow block crystals were obtained in 27% yield after 1 week. Selected IR peaks (cm^−1^): 2948 (w), 1656 (m), 1606 (w), 1420 (w), 1360 (w), 1241 (w), 1196 (m), 1130 (m), 1045 (w), 903 (w), 828 (w), 775 (s), 693 (s), 608 (m), 507 (m).

### Single crystal X-ray diffraction analyses

Single crystals of **SD/Ag51b**, **SD/Ag72a** and **SD/Ag72c** with appropriate dimensions were chosen under an optical microscope and quickly coated with high vacuum grease (Dow Corning Corporation) to prevent decomposition. Each crystal was mounted on CryoLoop™ loop and the cell parameters and intensity data were recorded on a Rigaku Oxford Diffraction XtaLAB Synergy-S diffractometer equipped with a HyPix-6000HE Hybrid Photon Counting (HPC) detector operating in shutterless mode and an Oxford Cryosystems Cryostream 800 Plus at 100 K using Cu Kα (λ = 1.54184 Å) for **SD/Ag51b** and **SD/Ag72c** and Mo Kα (λ = 0.71073 Å) for **SD/Ag72a** from PhotonJet micro-focus X-ray Source. Data were processed using the *CrystAlis*^Pro^ software suite^50^. These structures were solved using the charge-flipping algorithm, as implemented in the program *SUPERFLIP*^51^ and refined by full-matrix least-squares techniques against *F*_o_^2^ using the SHELXL program^52^ through the OLEX2 interface^53^. Hydrogen atoms at carbon were placed in calculated positions and refined isotropically by using a riding model. Appropriate restraints or constraints were applied to the geometry and the atomic displacement parameters of the atoms in the cluster. All structures were examined using the Addsym subroutine of PLATON^54^ to ensure that no additional symmetry could be applied to the models. Pertinent crystallographic data collection and refinement parameters are collated in Supplementary Table 4. Selected bond lengths and angles are collated in Supplementary Data 2.

### Experiment details

*i*PrSH (Adamas-beta®) and CF_3_COOAg (Adamas-beta^®^) were purchased from Shanghai Titan Scientific Co., Ltd. All other chemicals and solvents used in the syntheses were of analytical grade and used without further purification. IR spectra were recorded on a Perkin Elmer Spectrum Two in the frequency range of 4000–450 cm^−1^. Powder X-ray diffraction (PXRD) patterns were recorded for microcrystalline powdered samples using a Rigaku Oxford Diffraction XtaLAB Synergy-S diffractometer using Cu radiation (λ = 1.54184 Å). The PXRD patterns were processed with the *CrysAlis*^*Pro*^ software suite using the Powder function^50^. ^31^P NMR spectra were recorded in J. Young NMR tube on Bruker Avance 300 MHz spectrometer. UV-Vis spectra were performed on UV−Vis spectrophotometer (Evolution 220, ISA-220 accessory, Thermo Scientific). Temperature-dependent photoluminescence measurements were carried out in an Edinburgh spectrofluorimeter (FLS920) coupled with an Optistat DN cryostat (Oxford Instruments), and the ITC temperature controller and a pressure gauge were used to realize the variable-temperature measurement in the range of 113–293 K. Spectra were collected at different temperatures after a 5 min homoiothermy. Time-resolved photoluminescence lifetime measurements were measured on Edinburgh spectrofluorimeter (FLS920) using a time-correlated single-photon counting technique. Mass spectra were recorded on an Agilent 6224 (Agilent Technologies, USA) ESI-TOF-MS spectrometer. Sample solutions are infused by a syringe pump at 240 μL/h. Data were acquired using the following settings: electrospray ionization in positive mode, capillary voltage was set at 3.5 kV (-) and fragmentor at 200 V. The nebulizer was set to 15 psi and the nitrogen drying gas was set to a flow rate of 4 L/min. Drying gas temperature was maintained at 150 °C. The data analyses of mass spectra were performed based on the isotope distribution patterns using Agilent MassHunter Workstation Data acquisition software (Version B.05.00). The reported *m/z* values represent monoisotopic mass of the most abundant peak within the isotope pattern. The HAADF-STEM experiments were carried out at 300 kV using Thermofisher Spectra 300 scanning transmission electron microsope with a probe Cs-corrector.

### Computational details

DFT calculations were performed with the Gaussian 16 suite of programs^55^. To reduce the computational cost, the ligand-simplified Ag_51_ cluster [(PW_9_O_34_)@Ag_51_(CH_3_S)_25_(CF_3_COO)_17_(HCONH_2_)_3_(H_2_O)_3_] was used as a model for **SD/Ag51b**. The ligand simplification by changing *i*PrS to CH_3_S was done from the crystal structure of **SD/Ag51b** with GaussView and the resulting model cluster was directly used for the subsequent TD-DFT calculation. The B3PW91 hybrid density functional^56^ was employed in the TD-DFT calculation. The Los Alamos valence double-zeta with Hay-Wadt ECPs (LanL2DZ) basis set^57^ containing relativistic effects was employed for Ag and W atoms, and 6–31G basis set was used for other non-metal atoms. A total of 300 singlet states were chosen and the root was set as 1. Data for orbital composition analysis with Mulliken partition are from Gaussian 16 calculations and further processed with Multiwfn software^58^. The most probable transitions were determined based on the oscillator strength values and weights. The optical absorption spectrum was convoluted with a Gaussian line shape with a half-width at half-height of 0.25 eV.

## Supplementary information

Supplementary Information

Description of Additional Supplementary Files

Supplementary Data 1

Supplementary Data 2

## Data Availability

The data supporting the findings reported herein can be found in the manuscript, its supplementary Information, or from the authors upon request. The time-dependent density functional theory calculations of **SD/Ag51b** and the selected bond lengths (Å) and angles (^o^) for silver nanoclusters are provided with Supplementary Data 1 and 2, respectively. The X-ray crystallographic coordinates for structures reported in this article have been deposited at the Cambridge Crystallographic Data Centre, under deposition number CCDC: 2091982–2091984 for **SD/Ag51b**, **SD/Ag72a**, and **SD/Ag72c**. These data can be obtained free of charge from the Cambridge Crystallographic Data Centre via www.ccdc.cam.ac.uk/data_request/cif.
