# Supplementary material for: Nuclearity enlargement from [PW9O34@Ag51] to [(PW9O34)2@Ag72] and 2D and 3D network formation driven by bipyridines
Source: Nat Commun. 2022 Apr 4;13:1802. doi: 10.1038/s41467-022-29370-w (PMC8979969; doi:10.1038/s41467-022-29370-w)
Supplement: Supplementary file 1 — Supplementary Information [file 41467_2022_29370_MOESM1_ESM.pdf]

Supplementary Information

Nuclearity Enlargement from [PW_9O_{34}@Ag_{51}] to [(PW_9O_{34})$_2$@Ag_{72}] and 2D and 3D Network Formation Driven by Bipyridines

Zhi Wang,[1] Yan-Jie Zhu,[1] Ying-Zhou Li,[2] Gui-Lin Zhuang,[3] Ke-Peng Song,[1] Zhi-Yong Gao,[4] Jian-Min Dou,[5] Mohamedally Kurmoo,[6] Chen-Ho Tung,[1] and Di Sun[1*]

[1]School of Chemistry and Chemical Engineering, and State Key Laboratory of Crystal Materials, Shandong University, Ji'nan, 250100, People's Republic of China.

[2]Shandong Provincial Key Laboratory of Molecular Engineering, Qilu University of Technology (Shandong Academy of Science), Ji'nan, 250353, People's Republic of China.

[3]College of Chemical Engineering and Materials Science, Zhejiang University of Technology, Hangzhou, 310032, People's Republic of China.

[4]School of Chemistry and Chemical Engineering, Henan Normal University, Xinxiang, 453007, People's Republic of China.

[5]Shandong Provincial Key Laboratory of Chemical Energy Storage and Novel Cell Technology, and School of Chemistry and Chemical Engineering, Liaocheng University, Liaocheng 252000, People's Republic of China.

[6]Université de Strasbourg, Institut de Chimie de Strasbourg, CNRS-UMR 7177, 4 rue Blaise Pascal, 67008 Strasbourg Cedex, France.

[*]To whom correspondence should be addressed.

E-mail: dsun@sdu.edu.cn

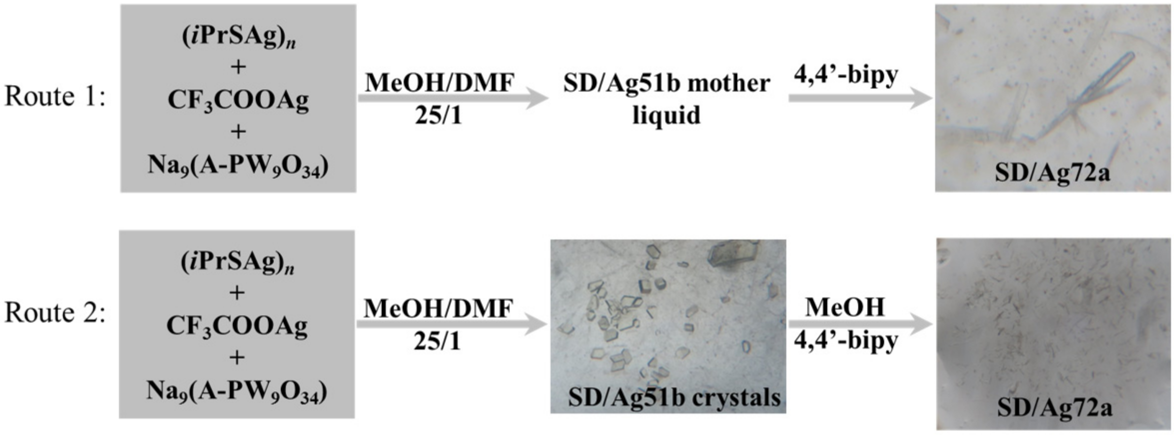

Supplementary Figure 1. Two transformation routes from SD/Ag51b to SD/Ag72a.

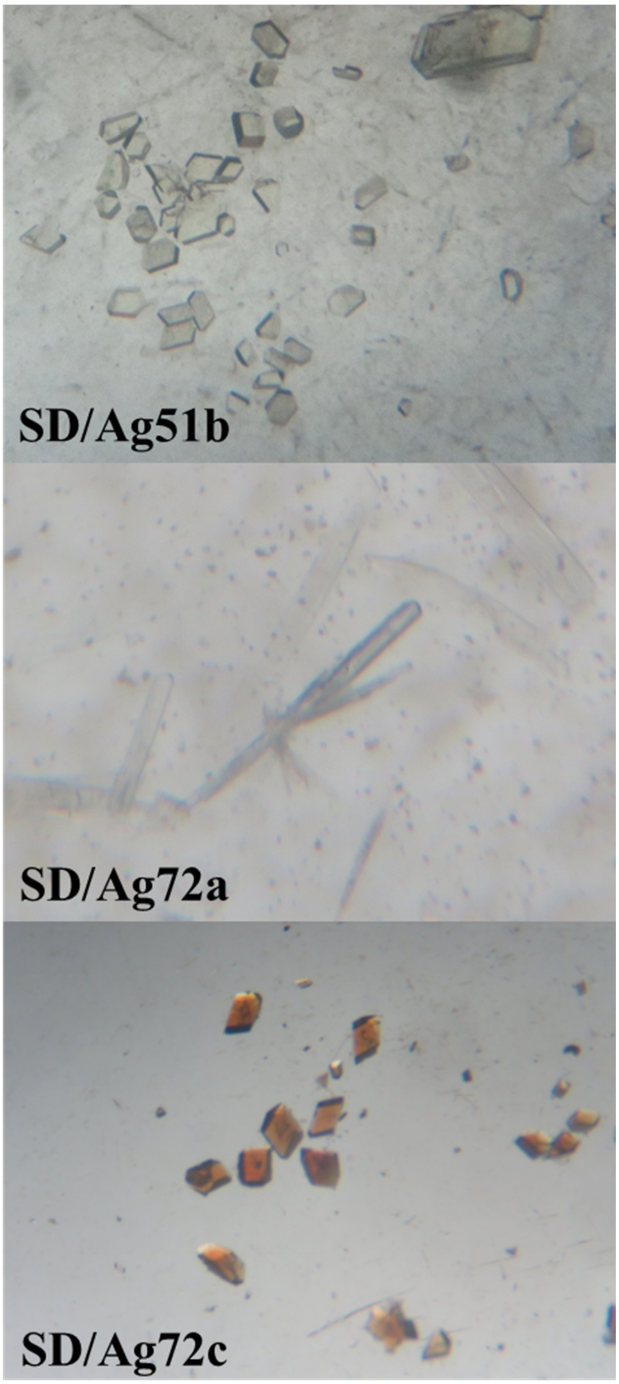

Supplementary Figure 2. Optical micrographs of crystals of SD/Ag51b, SD/Ag72a and SD/Ag72c.

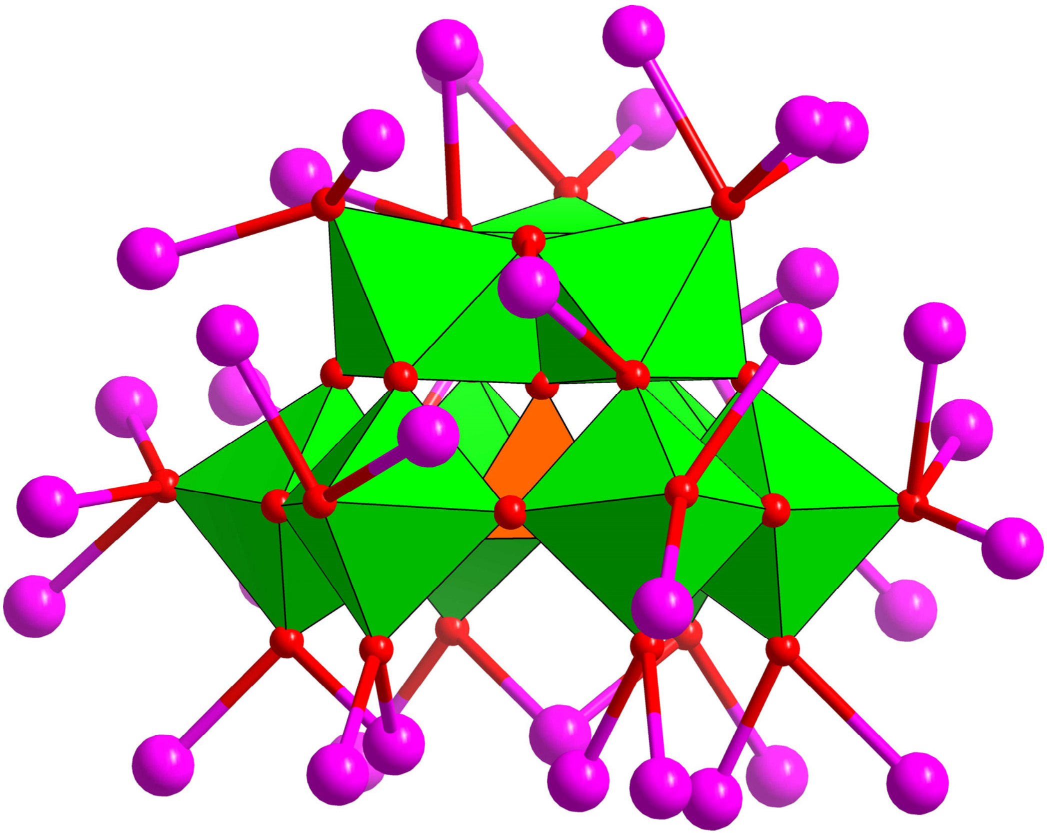

Supplementary Figure 3. The binding mode of $PW_9O_{34}^{9-}$ towards silver atoms in SD/Ag51b.

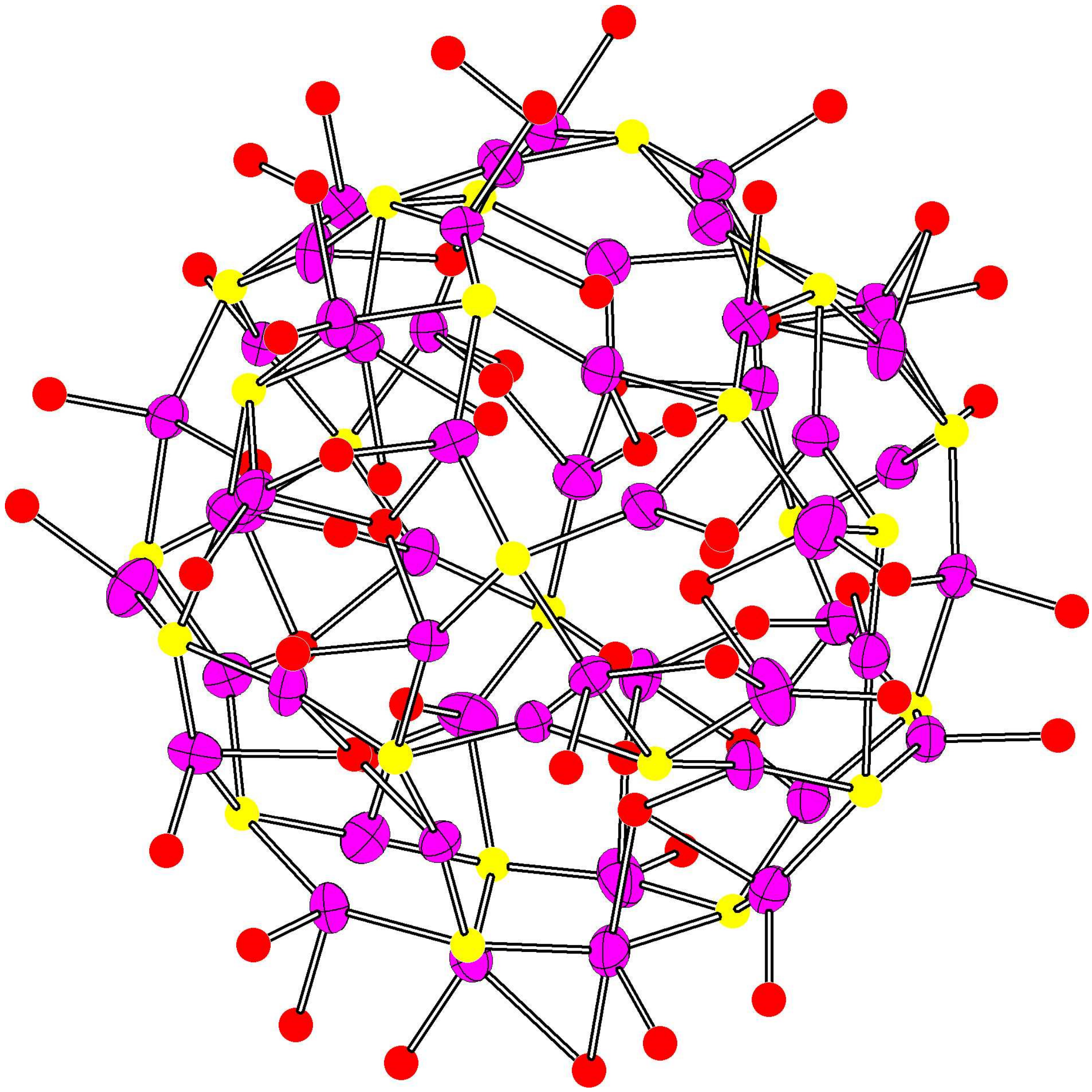

Supplementary Figure 4. The coordination spheres of silver atoms in SD/Ag51b. All non-coordinative atoms are removed for clarity. Color labels: purple, Ag; yellow, S; red, O.

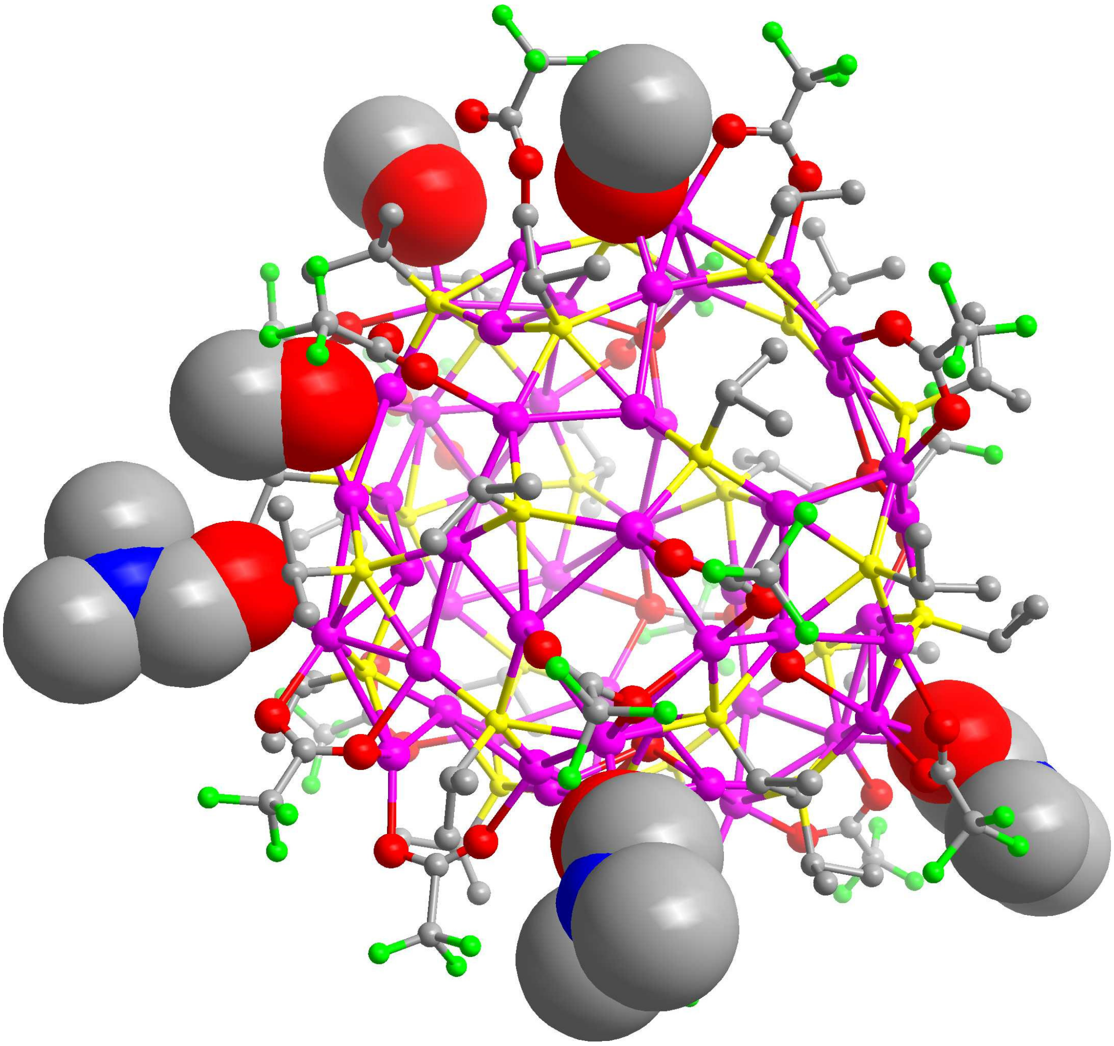

Supplementary Figure 5. The space-filling mode showing coordinated solvents on the surface of Ag_{51} shell. Color labels: purple, Ag; yellow, S; red, O; blue, N; green F; gray C.

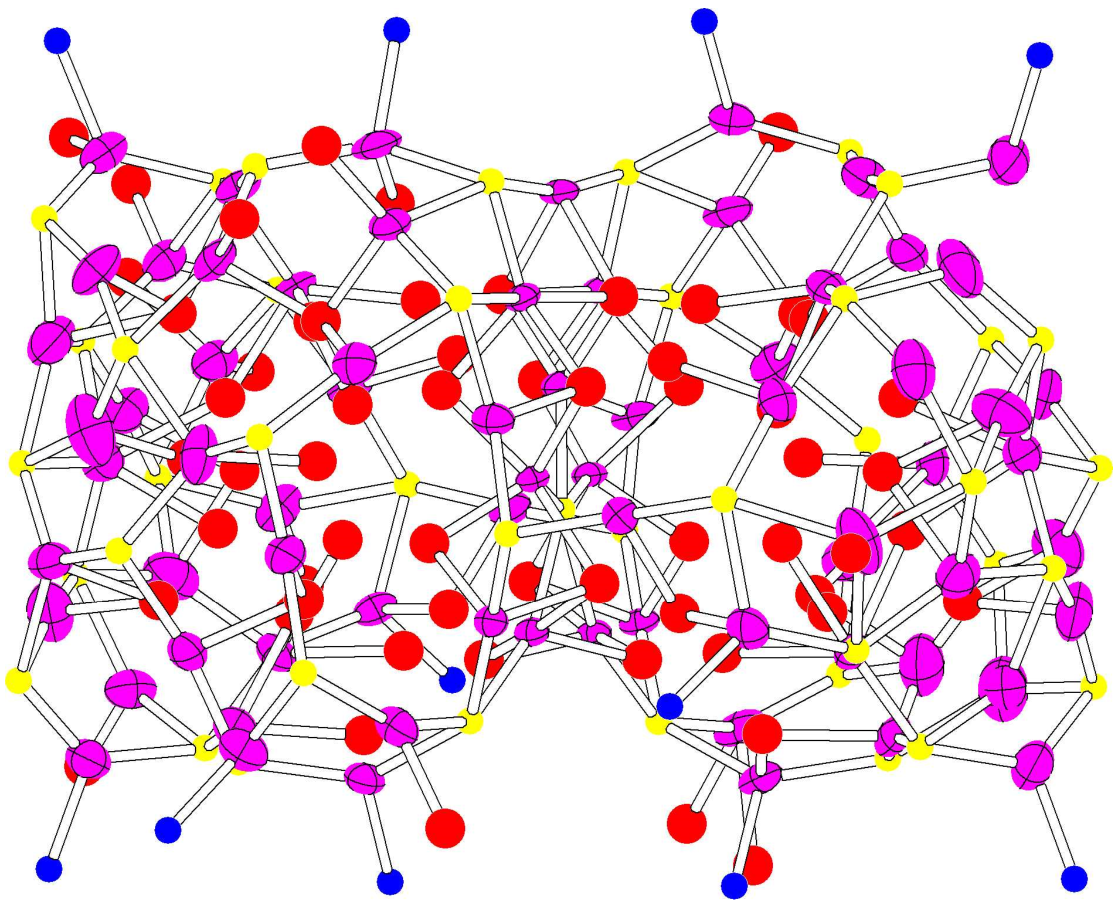

Supplementary Figure 6. The coordination spheres of silver atoms in SD/Ag72a. All non-coordinative atoms are removed for clarity. Color labels: purple, Ag; yellow, S; red, O; blue, N.

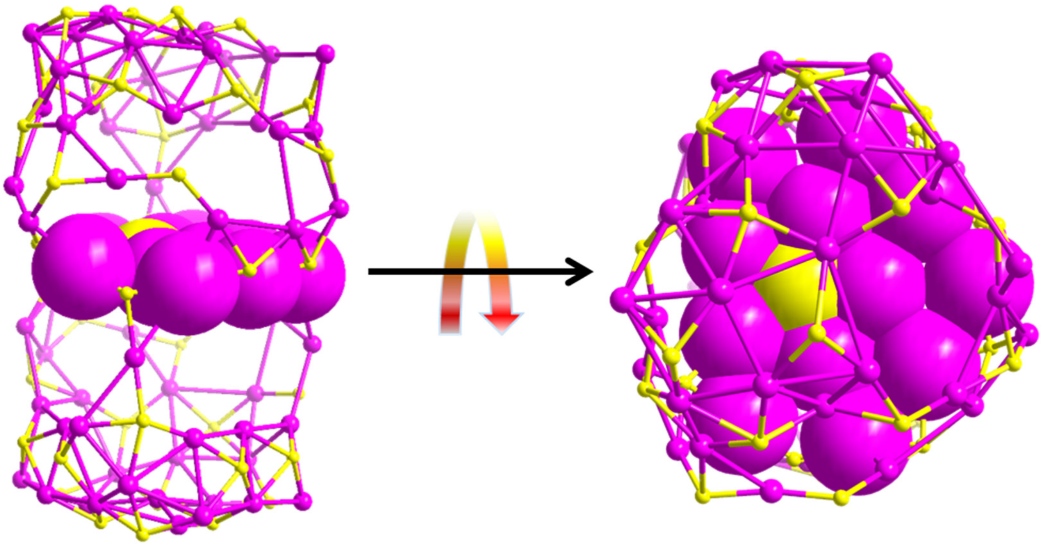

Supplementary Figure 7. The sandwich structure of Ag_{72} cluster with the equatorial S^{2-}-centered Ag_{12} plane shown in space-filling mode. Color labels: purple, Ag; yellow, S.

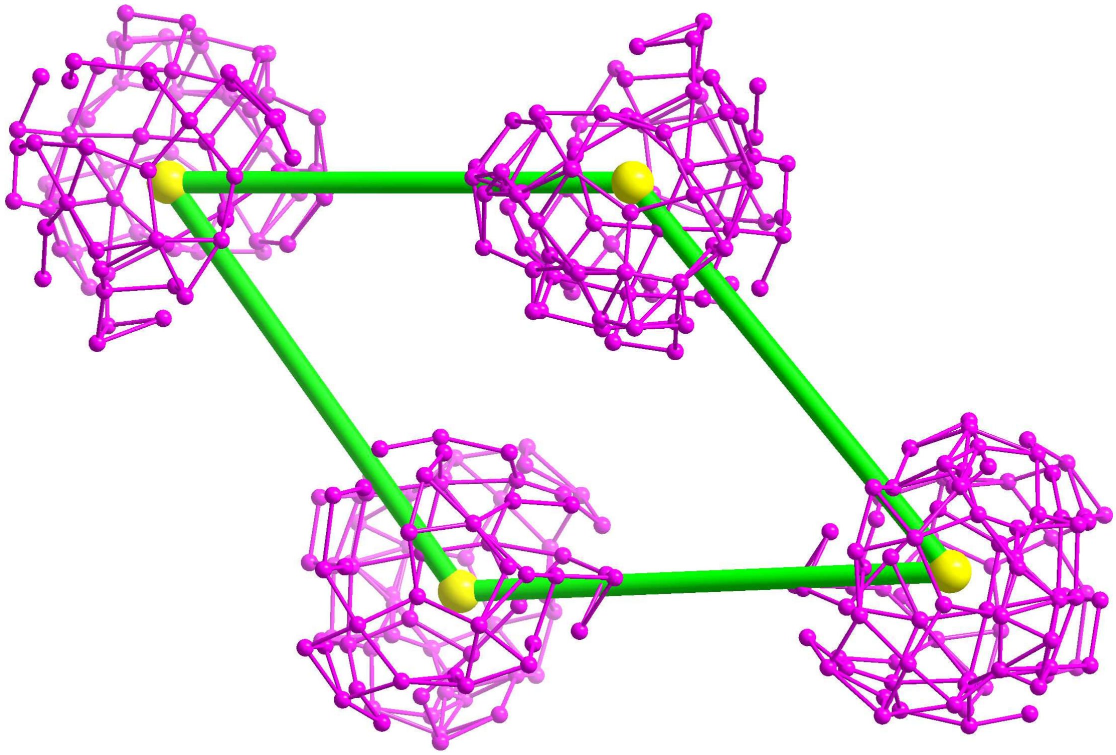

Supplementary Figure 8. The quadrilateral window in 2D 4^4-*sql* network with $[(PW_9O_{34})_2@Ag_{72}]$ as node and bipy as linker (green sticks).

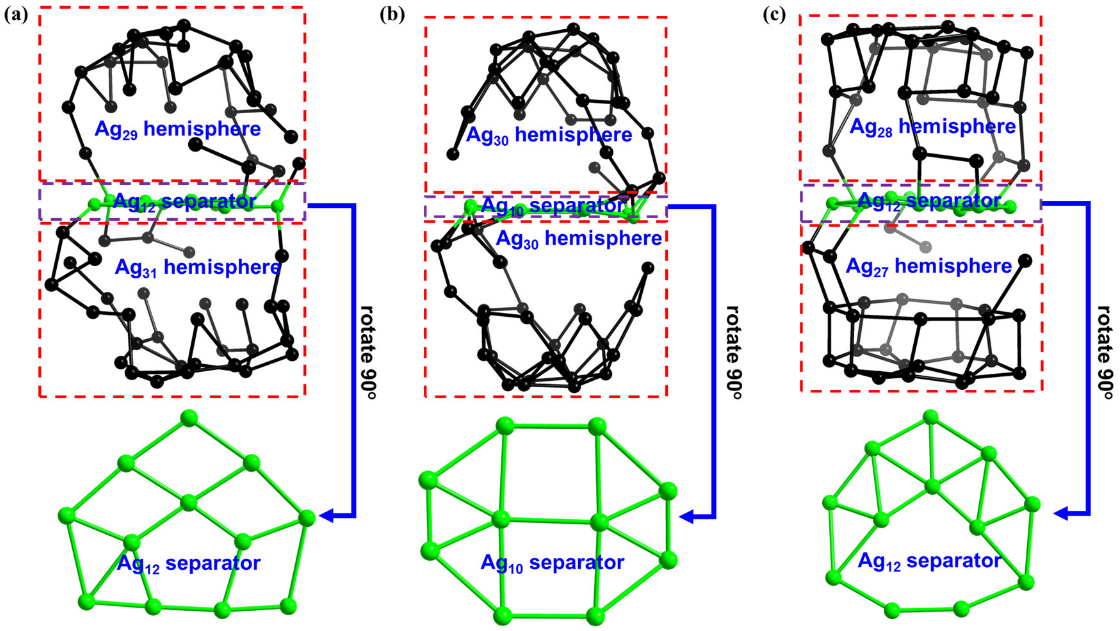

Supplementary Figure 9. The detailed comparisons of silver skeletons in SD/Ag72a (a), $[(PW_9O_{34})_2@Ag_{70}]$[1] (b) and $[(PW_9O_{34})_2@Ag_{67}]$[2] (c).

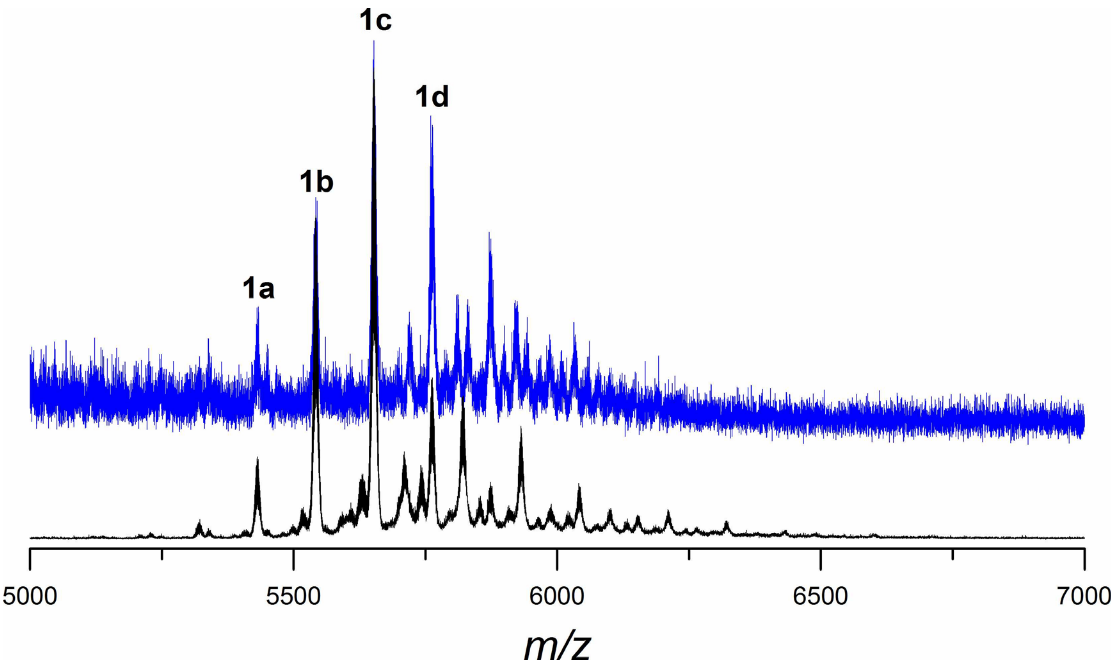

Supplementary Figure 10. The positive-ion mode ESI-MS of mother solution of SD/Ag51b (blue line) and SD/Ag51b dissolved in MeOH (black line).

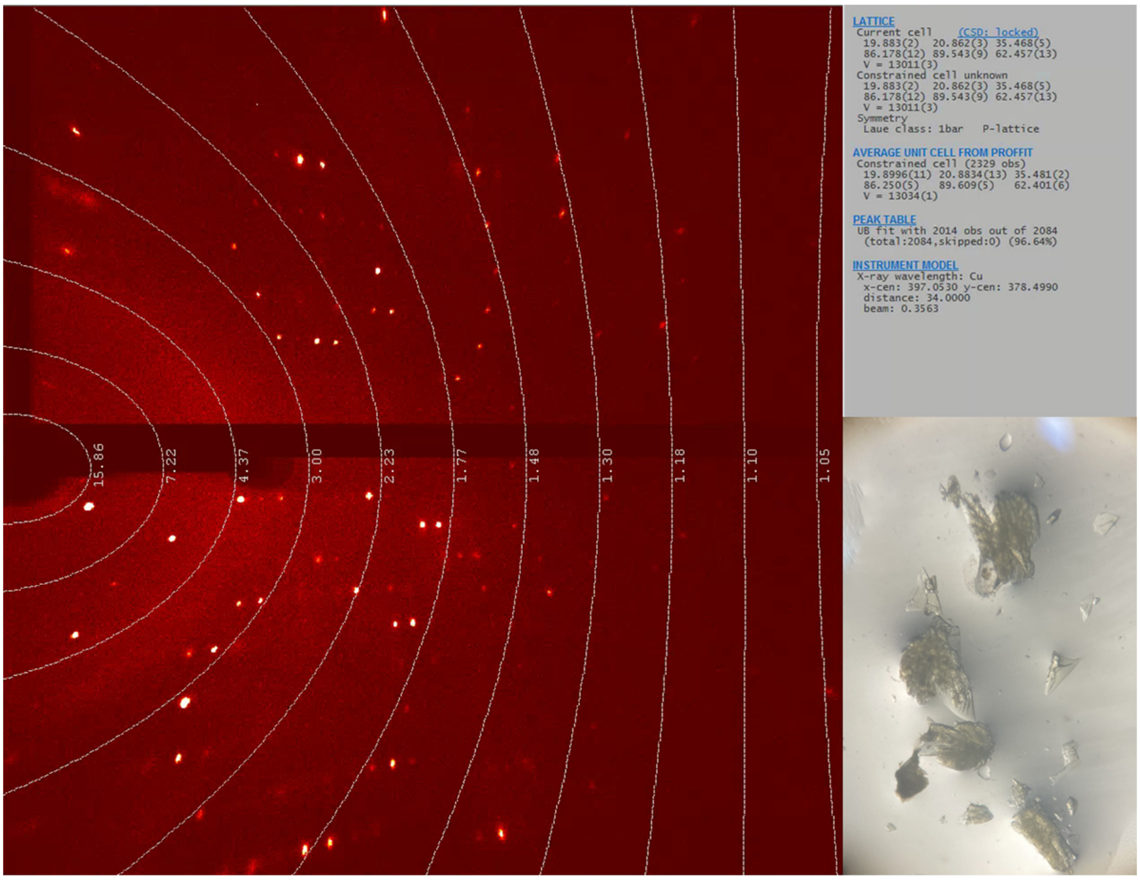

Supplementary Figure 11. Determination of cell parameters of crystals recrystallized from methanolic solution of SD/Ag51b. Inset at right bottom down: Optical micrograph of crystals recrystallized from methanolic solution of SD/Ag51b.

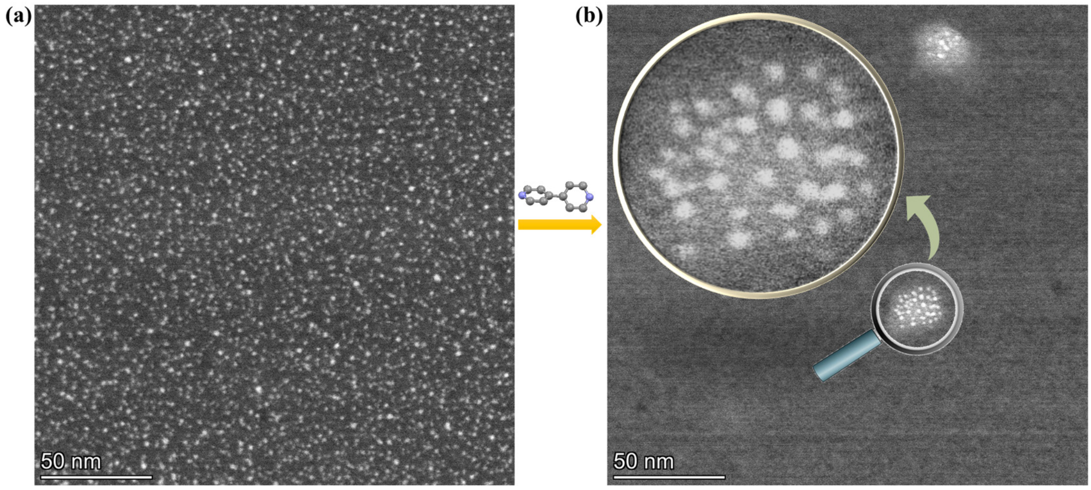

Supplementary Figure 12. HAADF-STEM images of methanol solution SD/Ag51b before (a) and after (b) adding bipy.

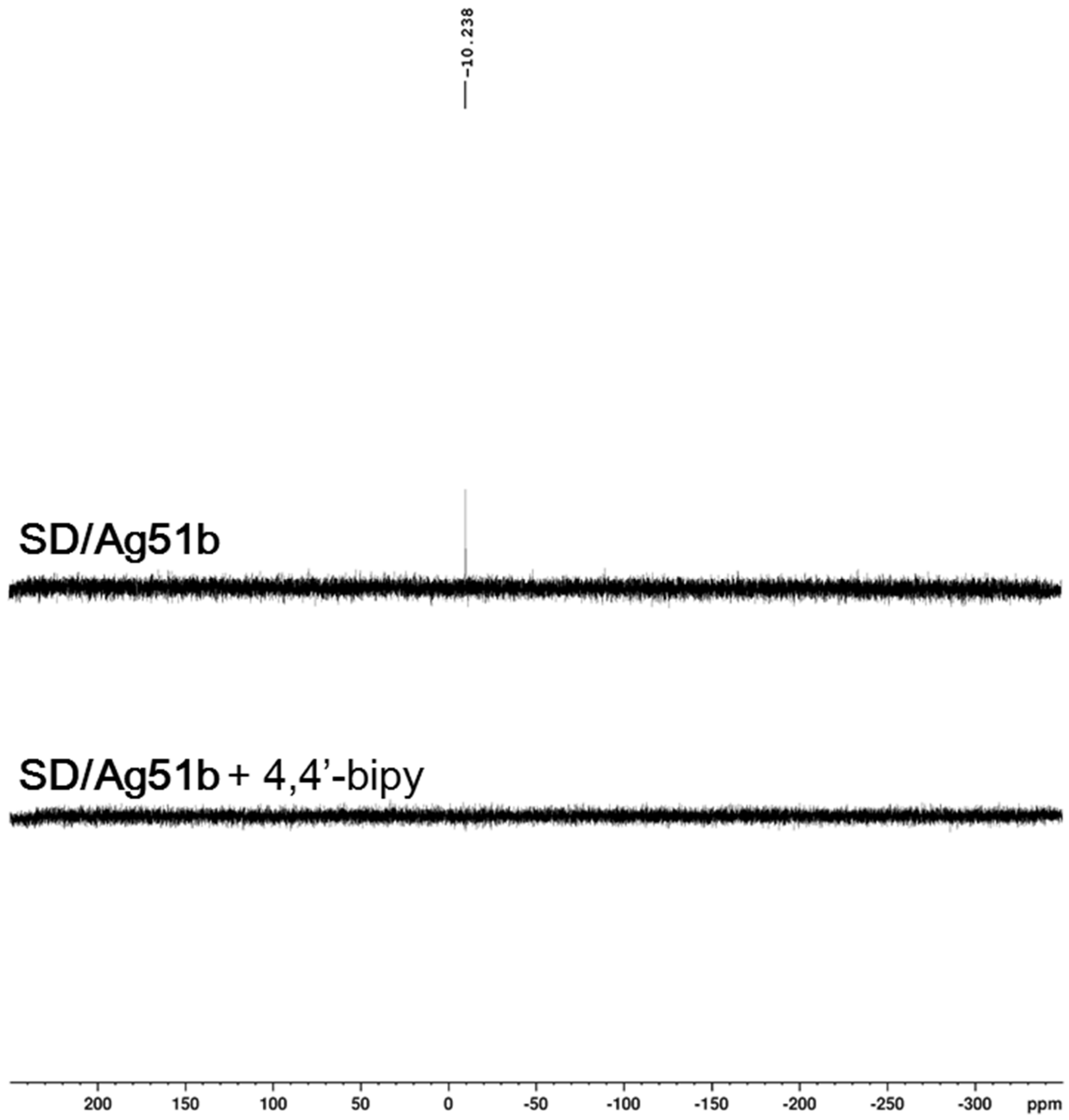

Supplementary Figure 13. The ^{31}P NMR spectra of SD/Ag51b before and after adding bipy.

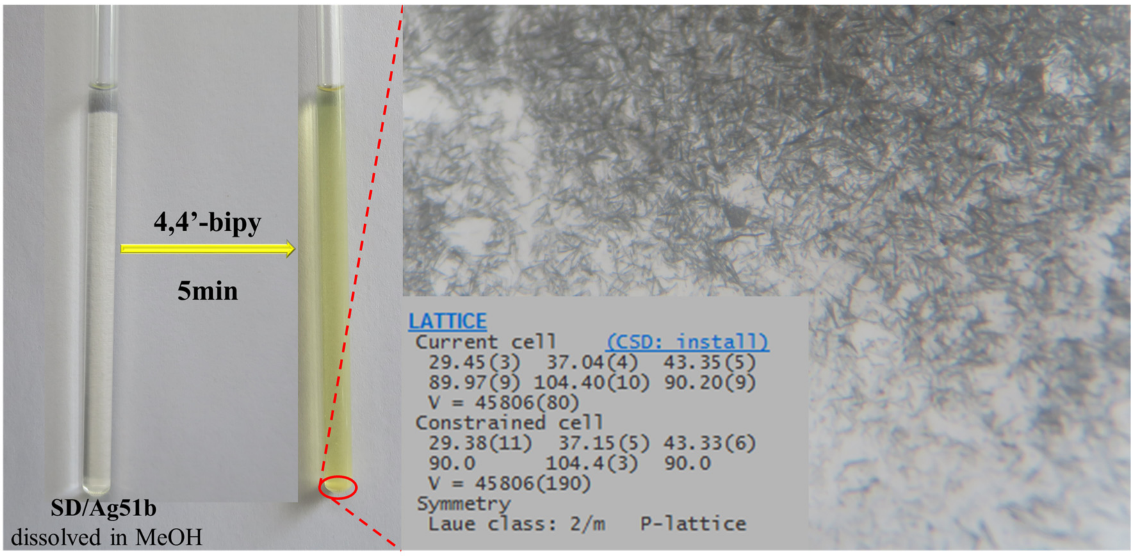

Supplementary Figure 14. The quick conversion from SD/Ag51b to SD/Ag72a in NMR tube.

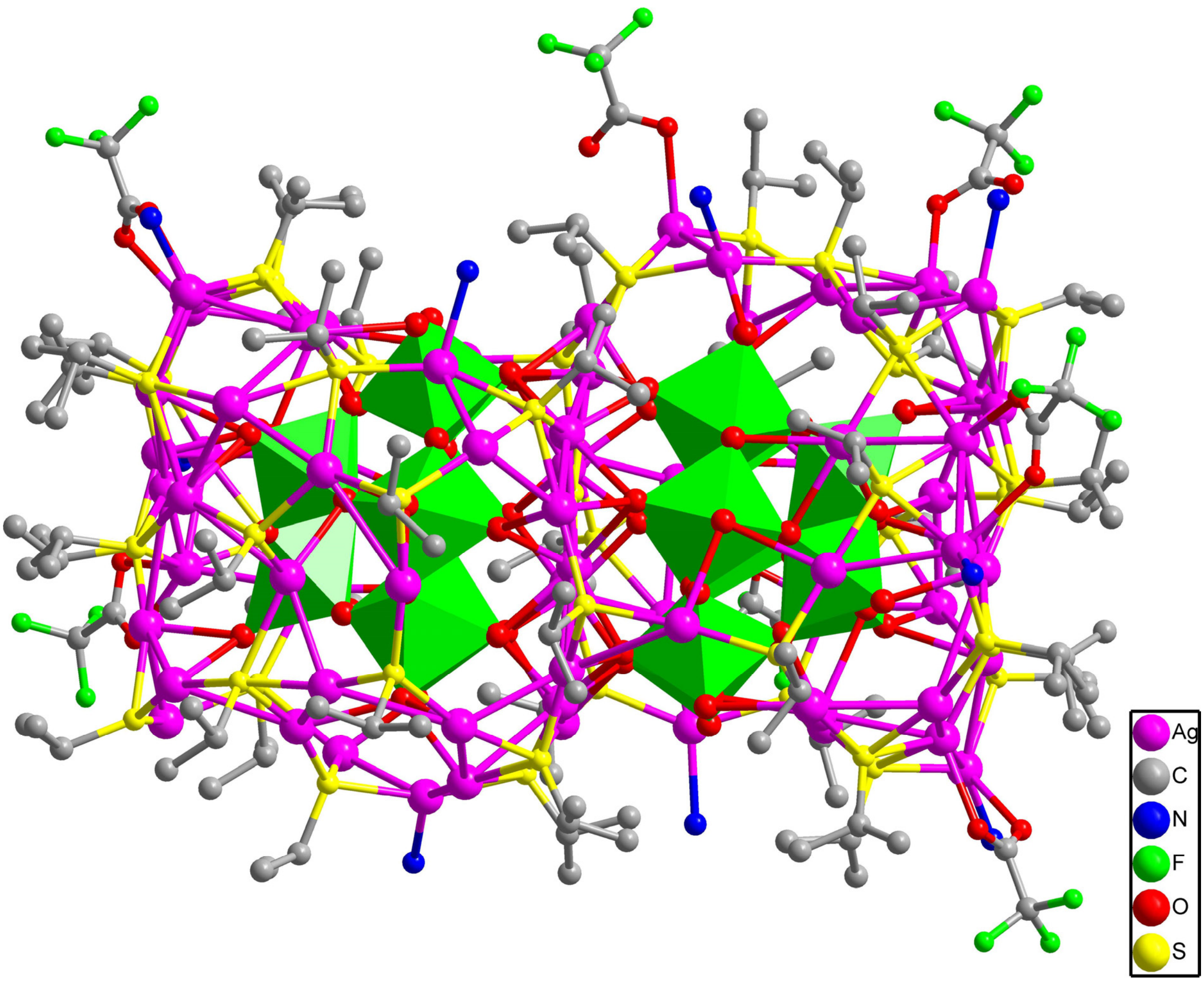

Supplementary Figure 15. The asymmetric unit of $(PW_9O_{34})_2$@Ag_{72} in SD/Ag72c. Only N atoms of coordinated pi-bipy are shown and all H atoms are omitted for clarity. $PW_9O_{34}^{9-}$ is shown in polyhedron mode with PO_4 and WO_6 colored as brown and green.

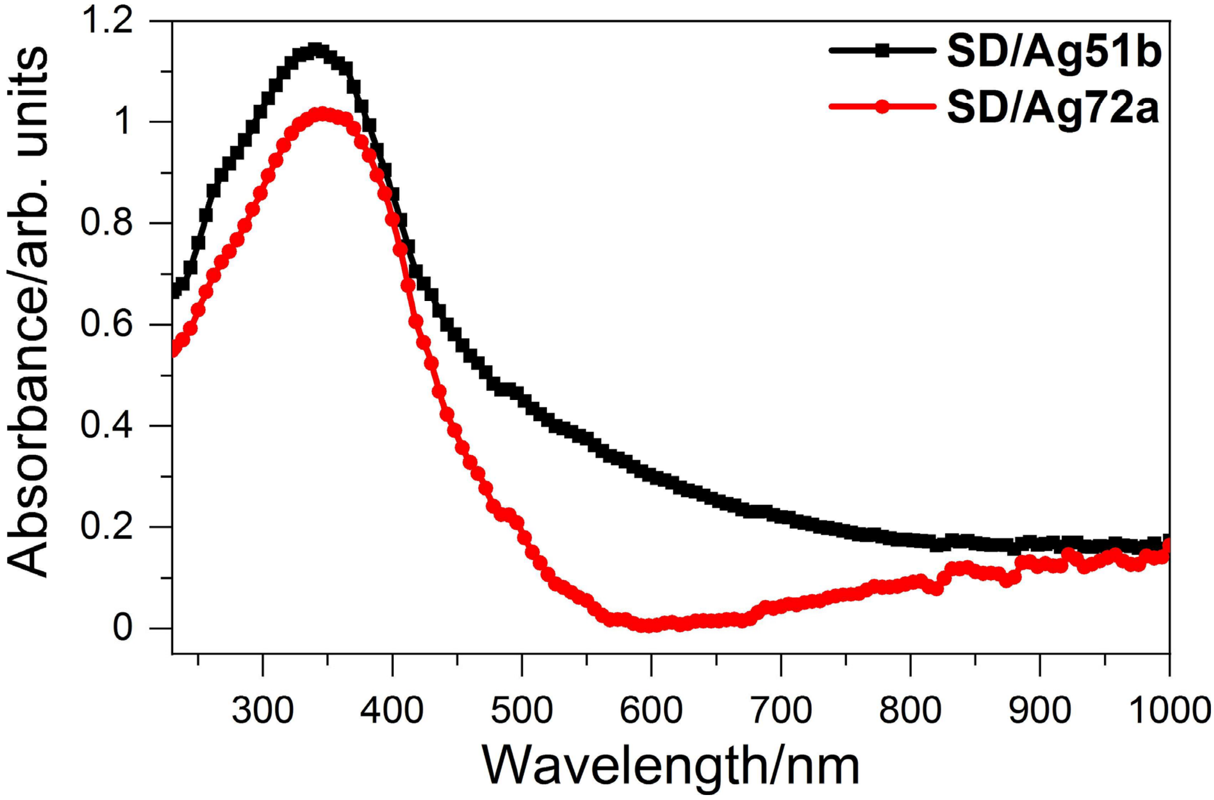

Supplementary Figure 16. The UV/Vis spectra of SD/Ag51b and SD/Ag72a.

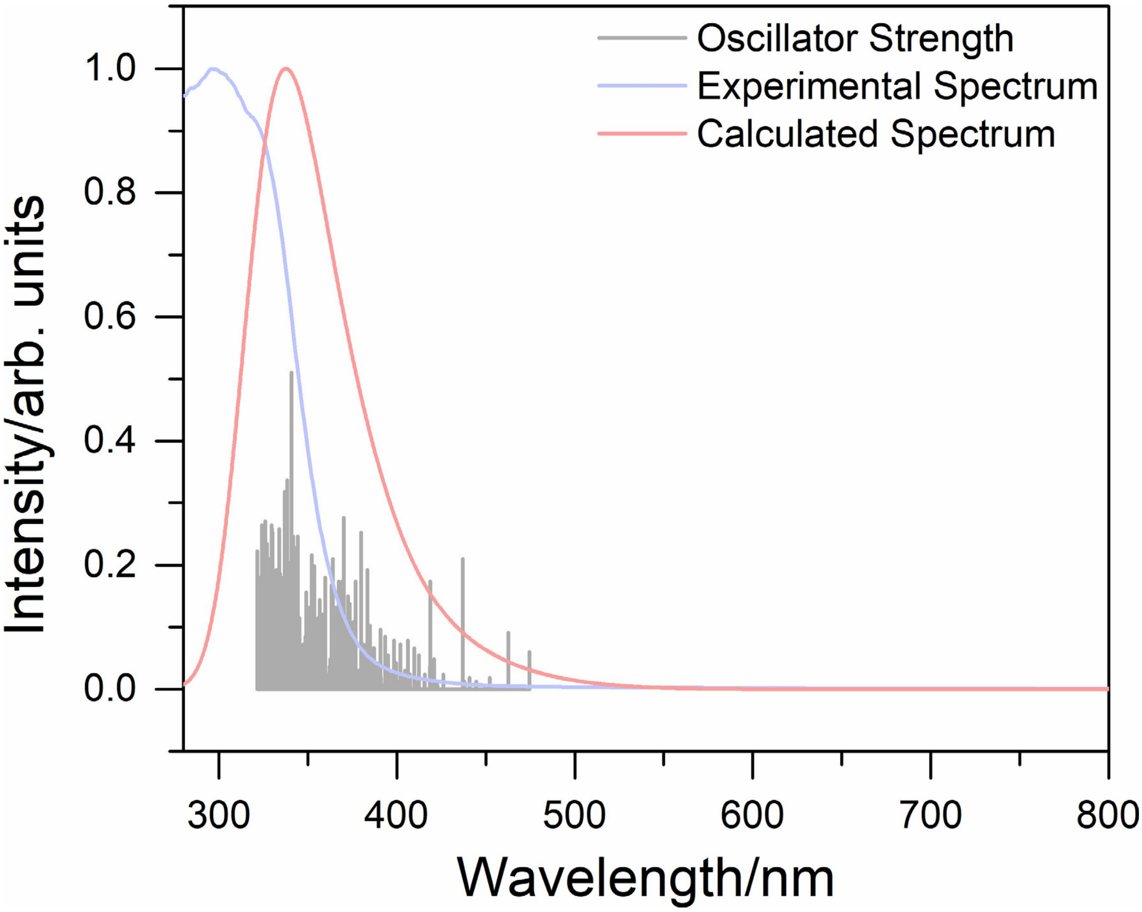

Supplementary Figure 17. The experimental (SD/Ag51d crystals dissolved in methanol) and TD-DFT calculated UV-Vis spectra of model SD/Ag51b.

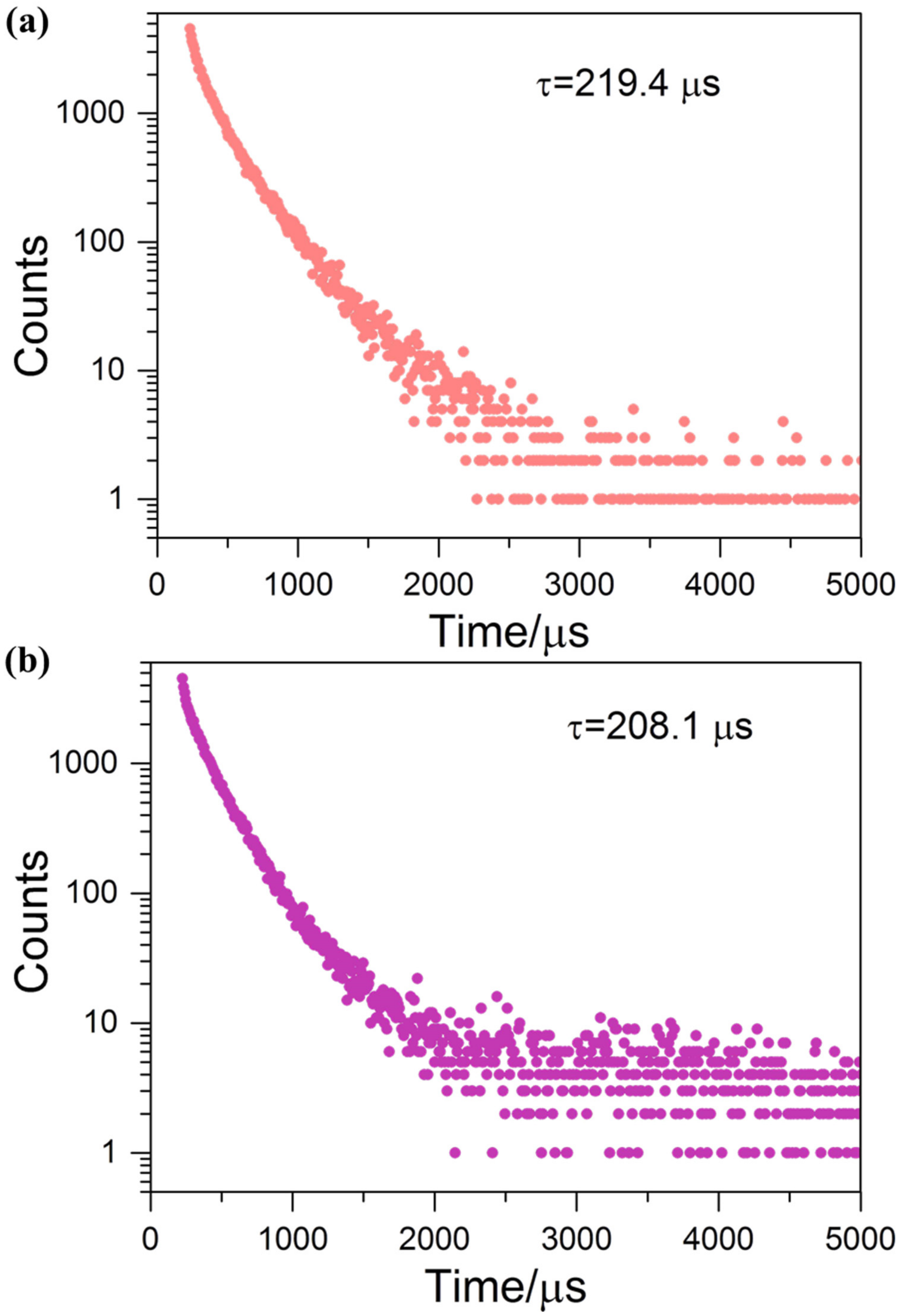

Supplementary Figure 18. Luminescent lifetimes of SD/Ag51b (a) and SD/Ag72a (b) at 113 K.

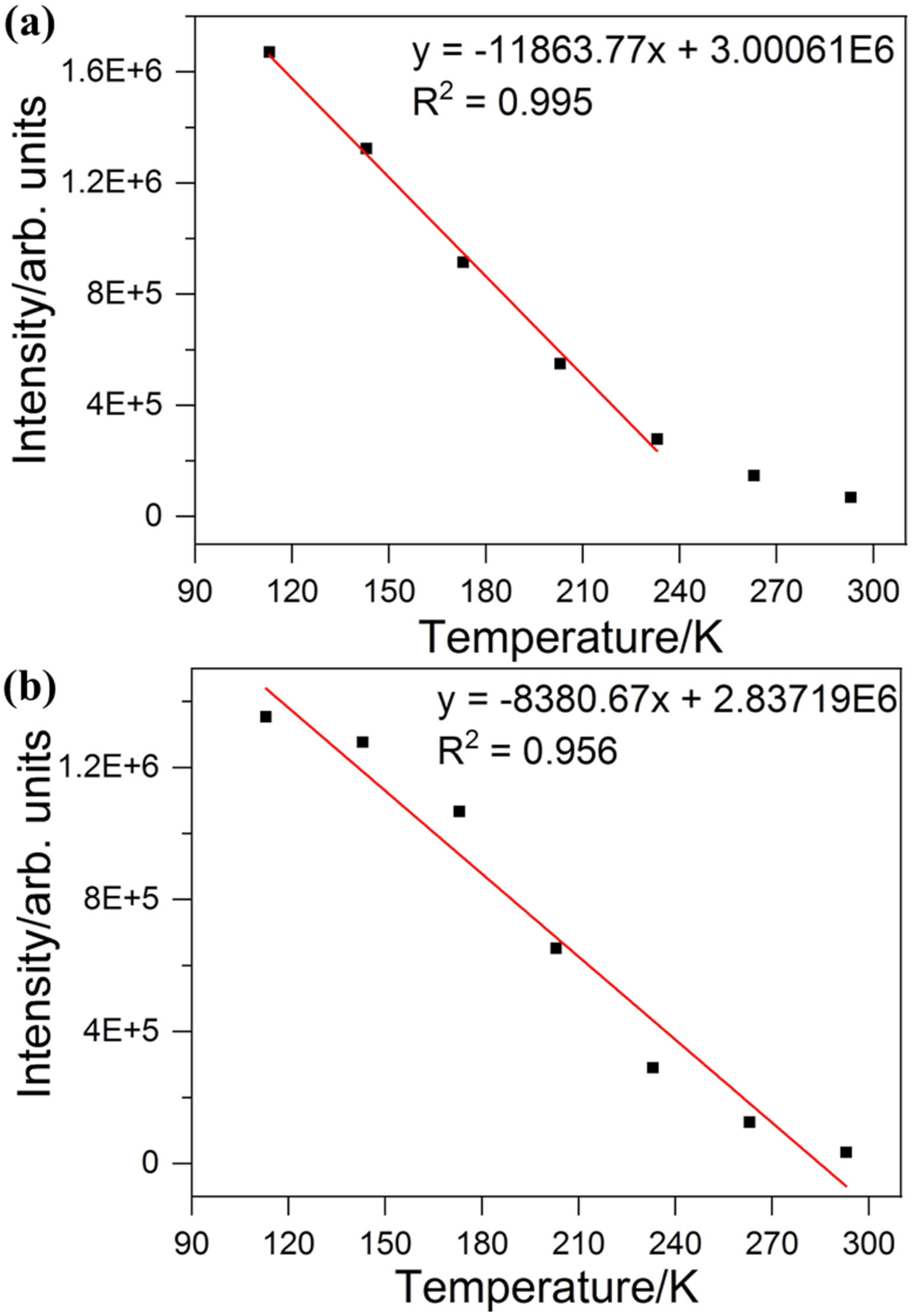

Supplementary Figure 19. The plot of temperature vs. maximum emission intensity of SD/Ag51b ((a), red line is the linear fitting in the range from 113 to 233 K) and SD/Ag72a ((b), red line is the linear fitting in the range from 113 to 293 K).

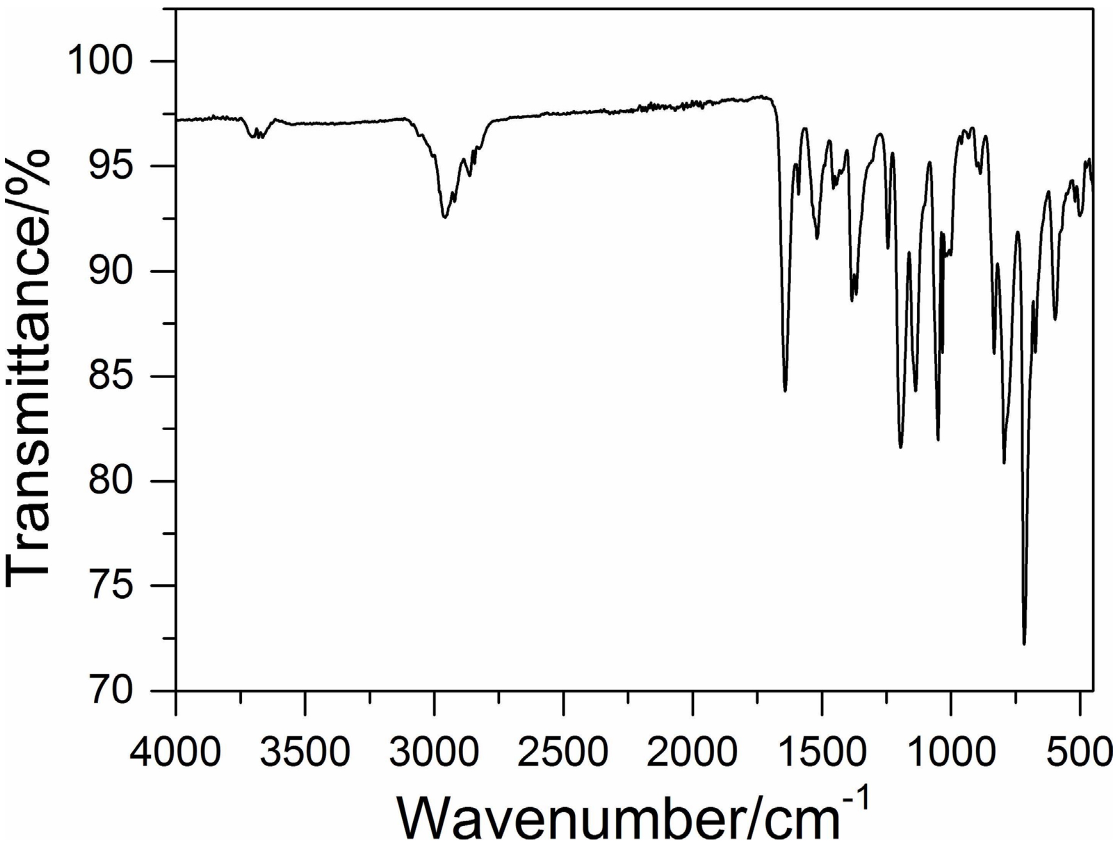

Supplementary Figure 20. The IR spectrum of SD/Ag51b.

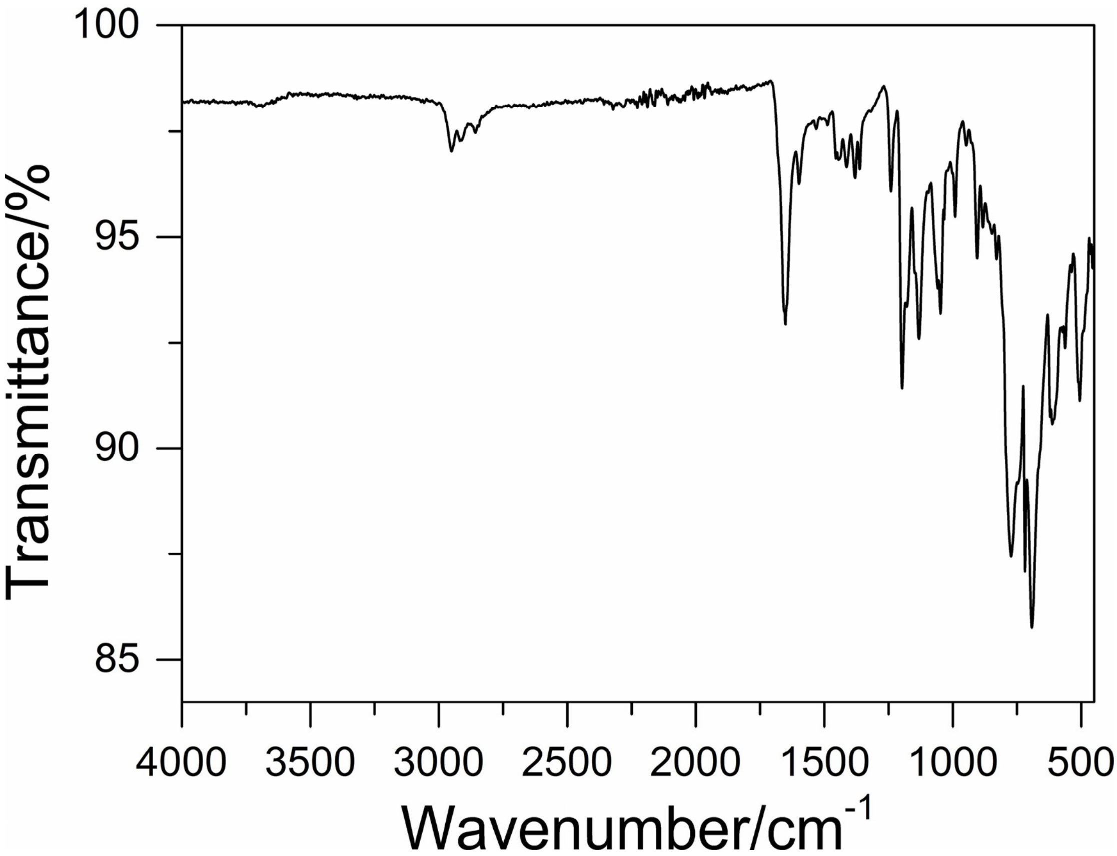

Supplementary Figure 21. The IR spectrum of SD/Ag72a.

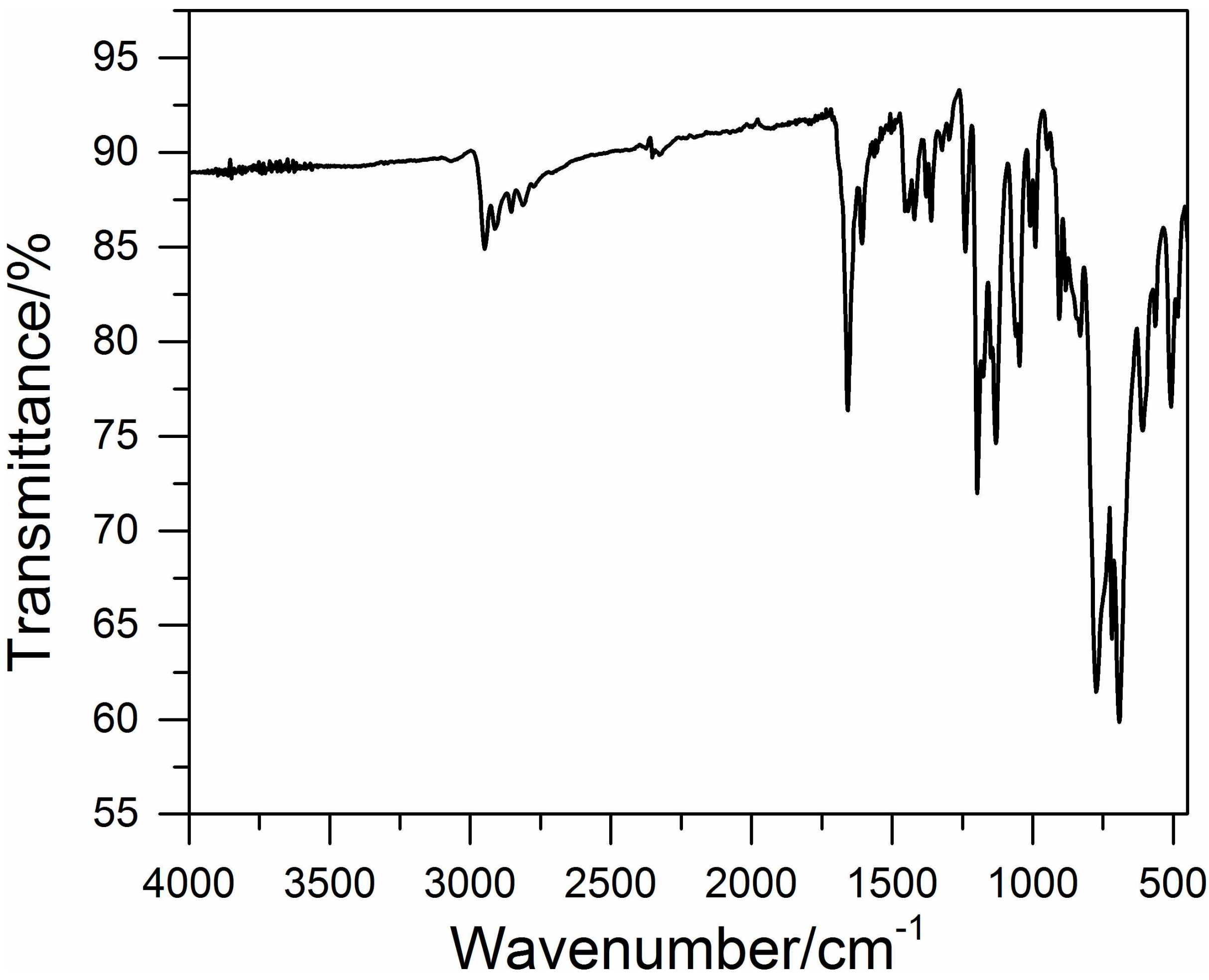

Supplementary Figure 22. The IR spectrum of SD/Ag72c.

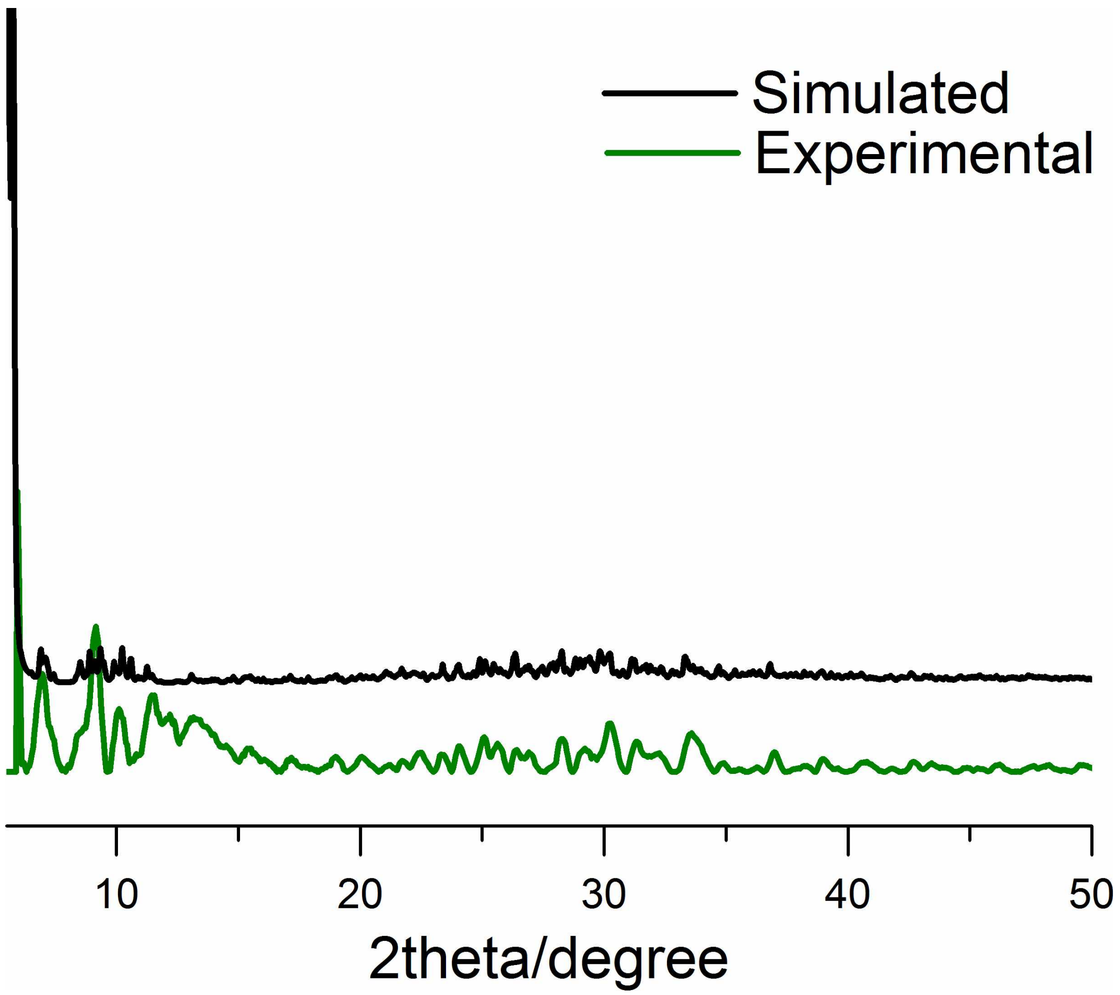

Supplementary Figure 23. Comparison of observed and simulated PXRD patterns of SD/Ag51b.

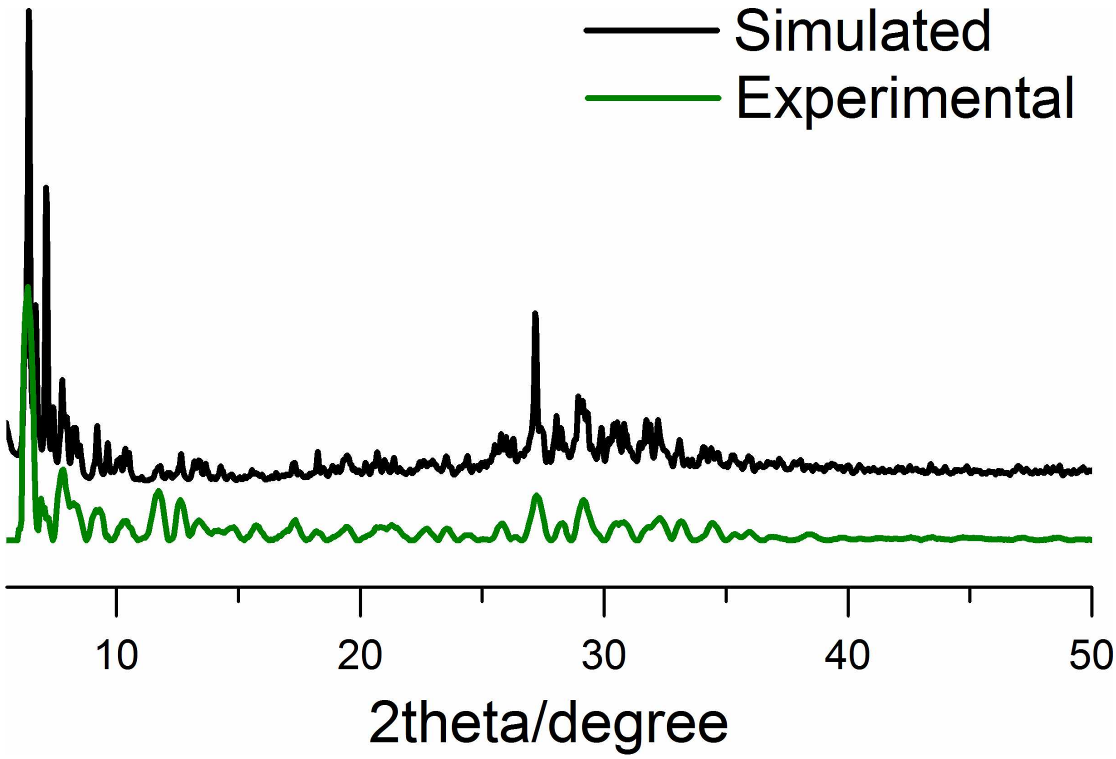

Supplementary Figure 24. Comparison of observed and simulated PXRD patterns of SD/Ag72a.

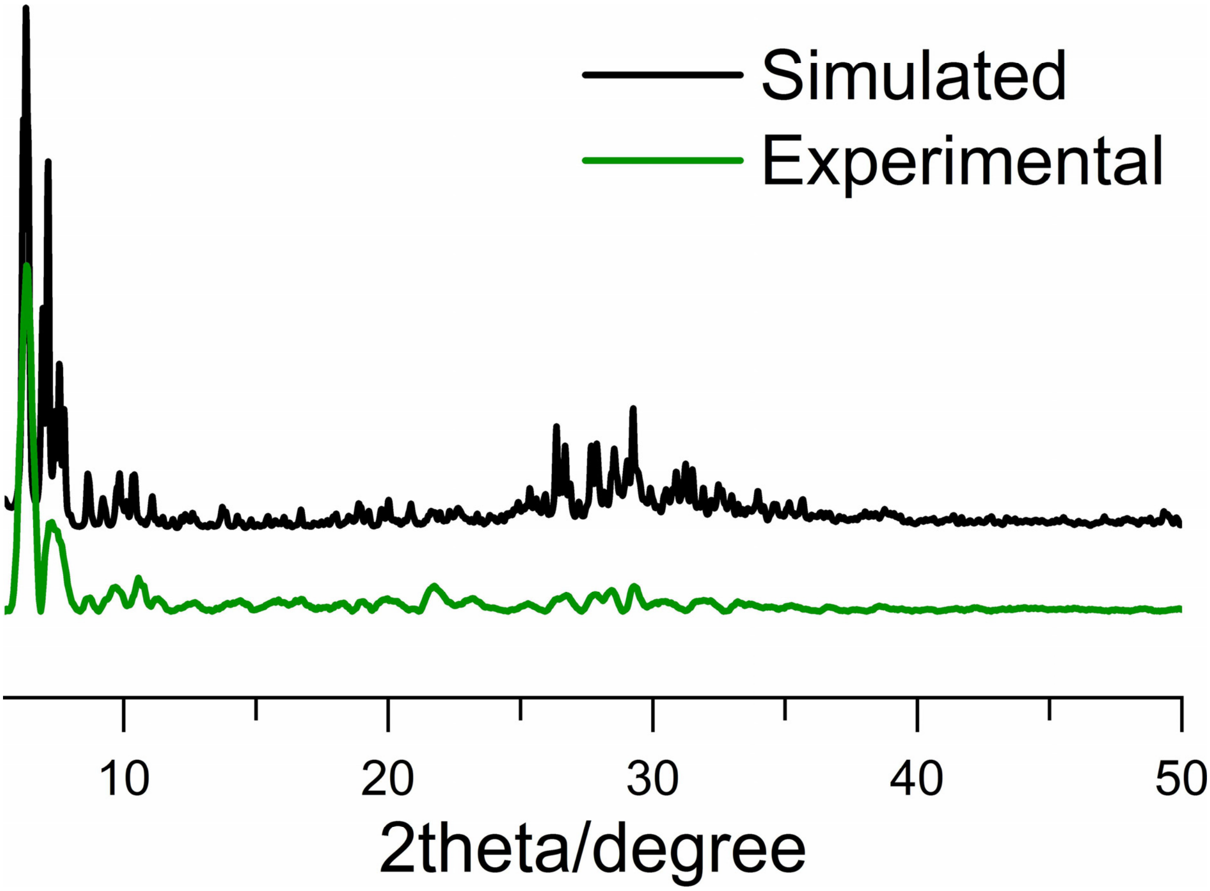

Supplementary Figure 25. Comparison of observed and simulated PXRD patterns of SD/Ag72c.

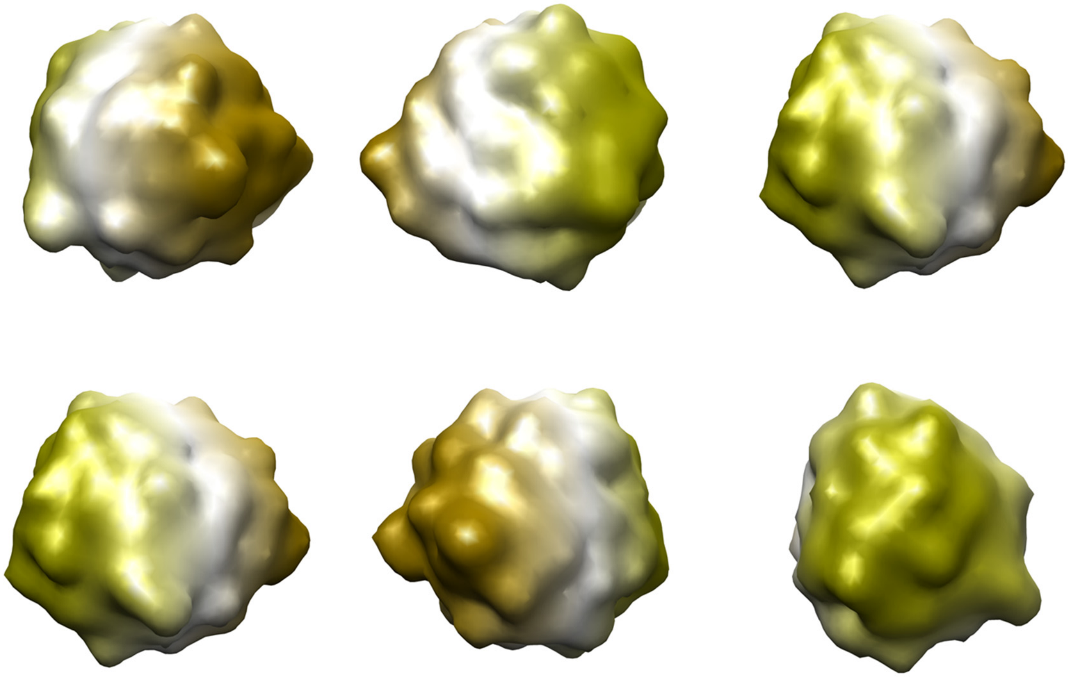

Supplementary Figure 26. The surface of SD/Ag51b calculated via 3V Volume Assessor program[3] by rolling a virtual probe (0.8 Å) on the surface viewed along six different orientations.

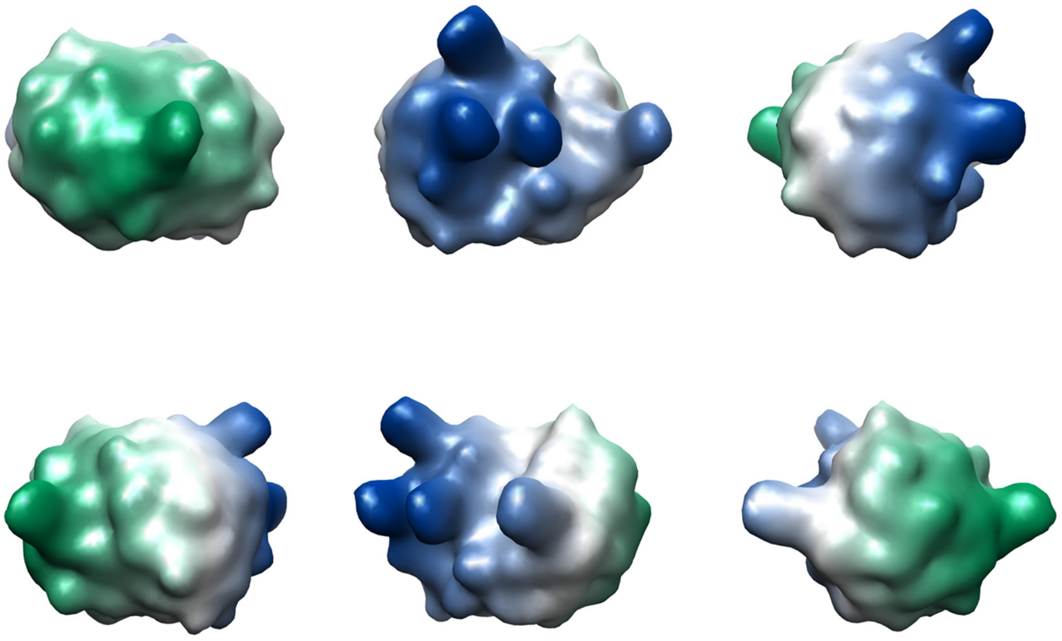

Supplementary Figure 27. The surface of SD/Ag72a unit calculated via 3V Volume Assessor program[3] by rolling a virtual probe (0.8 Å) on the surface viewed along six different orientations.

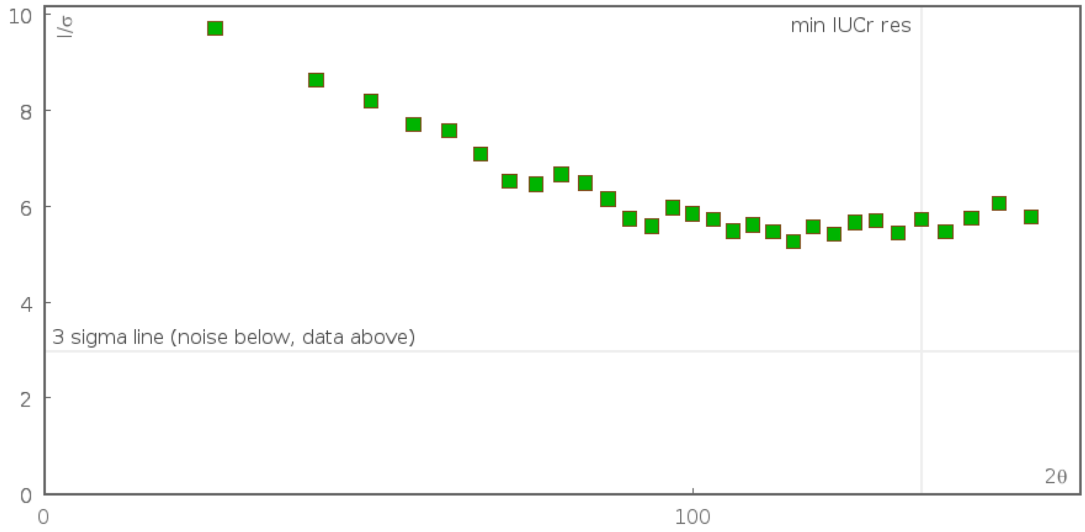

Supplementary Figure 28. The I/sigma vs. resolution plot of SD/Ag51b derived from reflection data statistics using OLEX 1.2.10.[4]

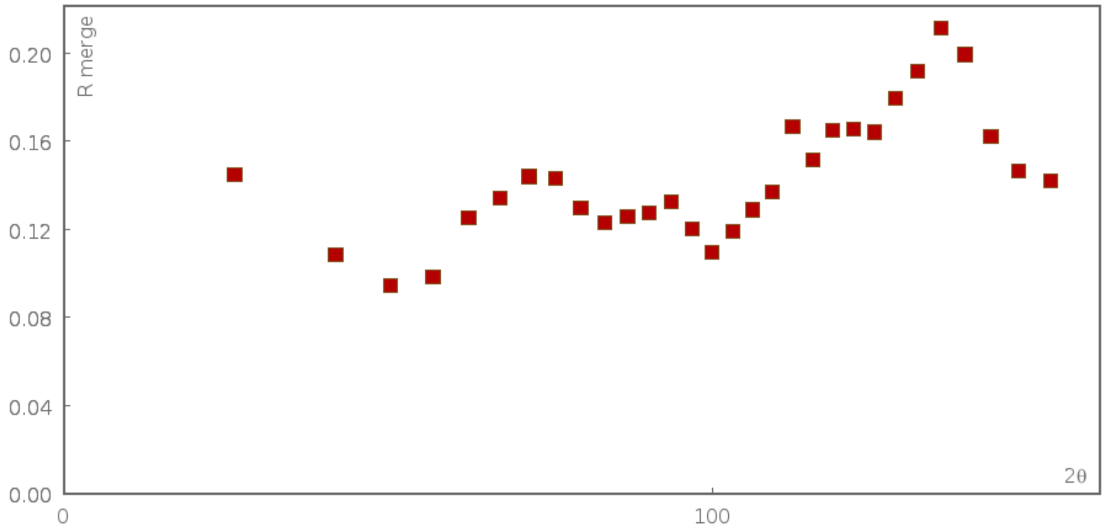

Supplementary Figure 29. The R_{merge} vs. resolution plot of SD/Ag51b derived from reflection data statistics using OLEX 1.2.10.[4]

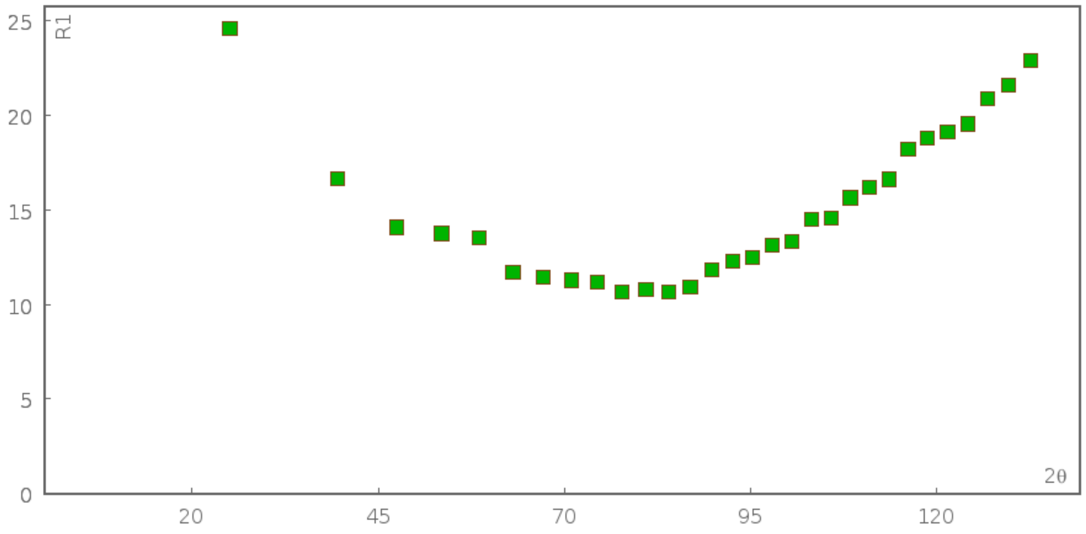

Supplementary Figure 30. The R_1 factor vs. resolution plot of SD/Ag51b derived from reflection data statistics using OLEX 1.2.10.[4]

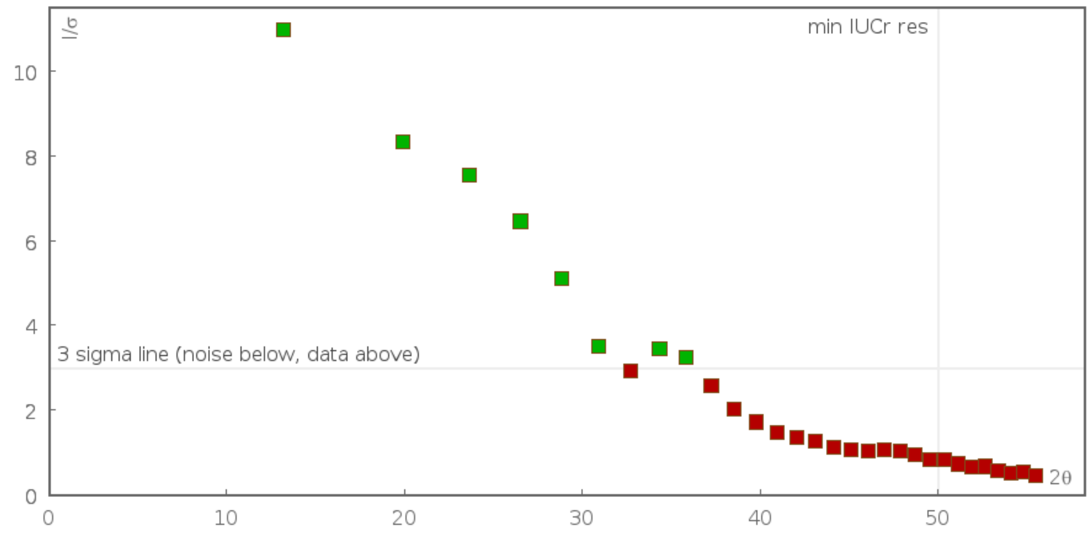

Supplementary Figure 31. The I/sigma vs. resolution plot of SD/Ag72a derived from reflection data statistics using OLEX 1.2.10.[4]

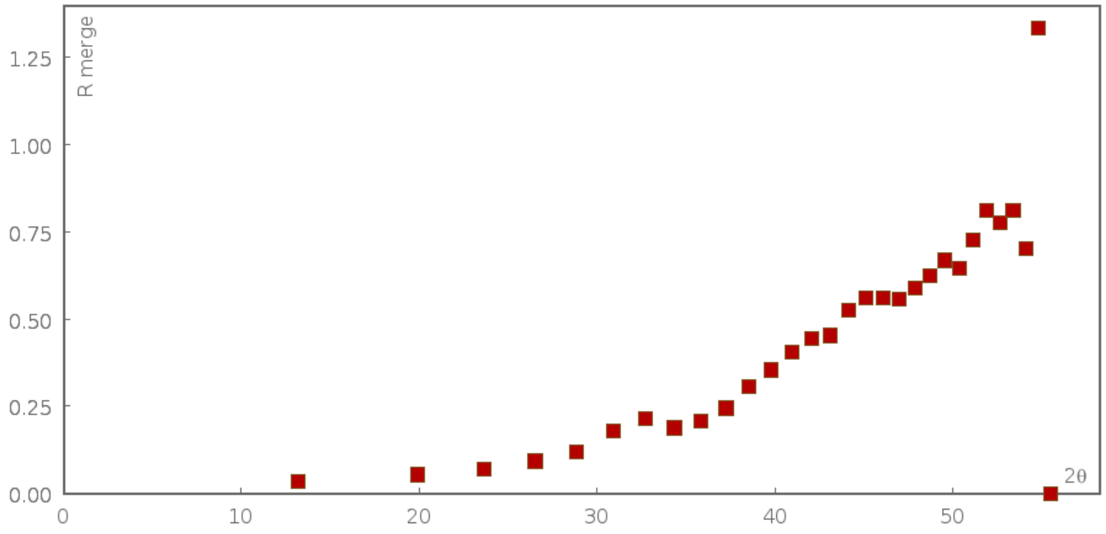

Supplementary Figure 32. The R_{merge} vs. resolution plot of SD/Ag72a derived from reflection data statistics using OLEX 1.2.10.[4]

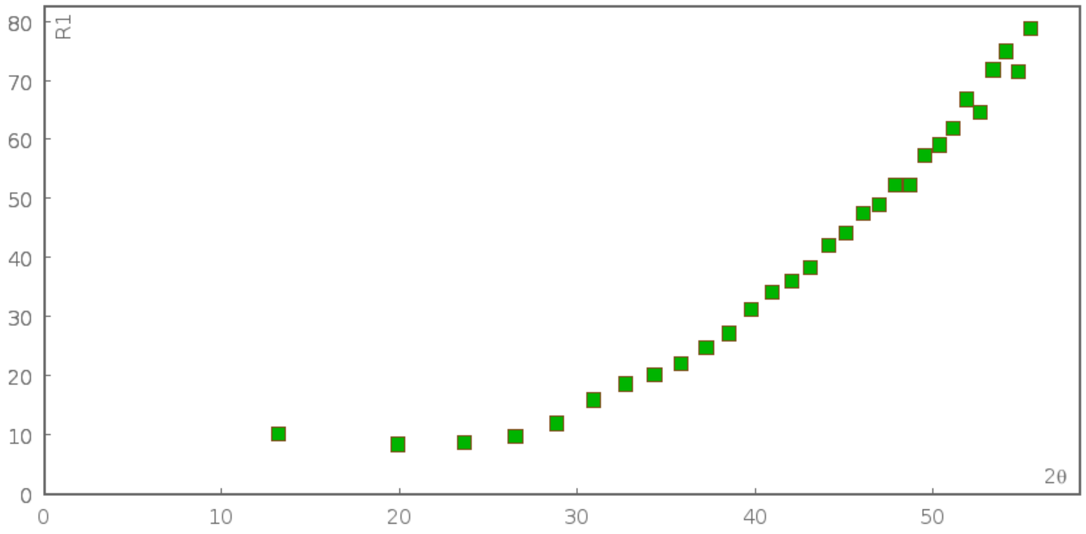

Supplementary Figure 33. The R_1 factor vs. resolution plot of SD/Ag72a derived from reflection data statistics using OLEX 1.2.10.[4]

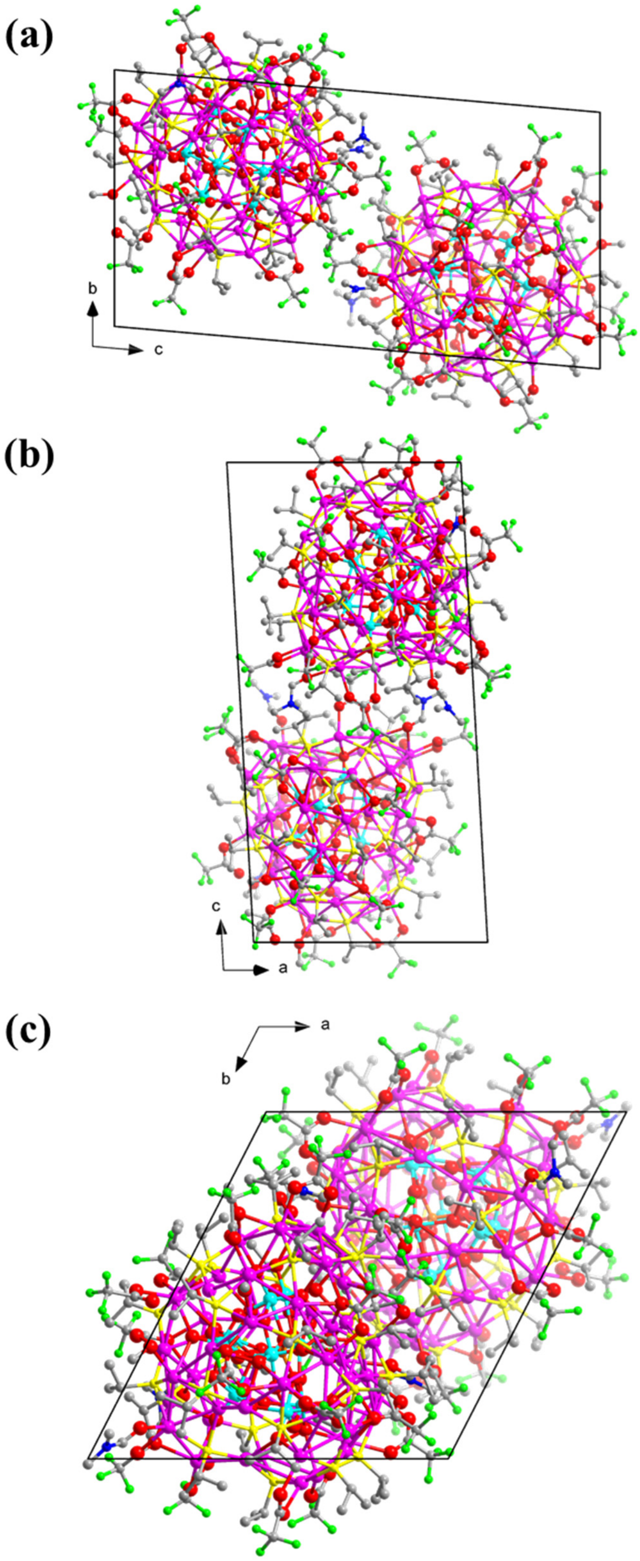

Supplementary Figure 34. Molecules packing diagrams in 1×1×1 unit cell of SD/Ag51b viewed along *a* (a), *b* (b), *c* (c) axis.

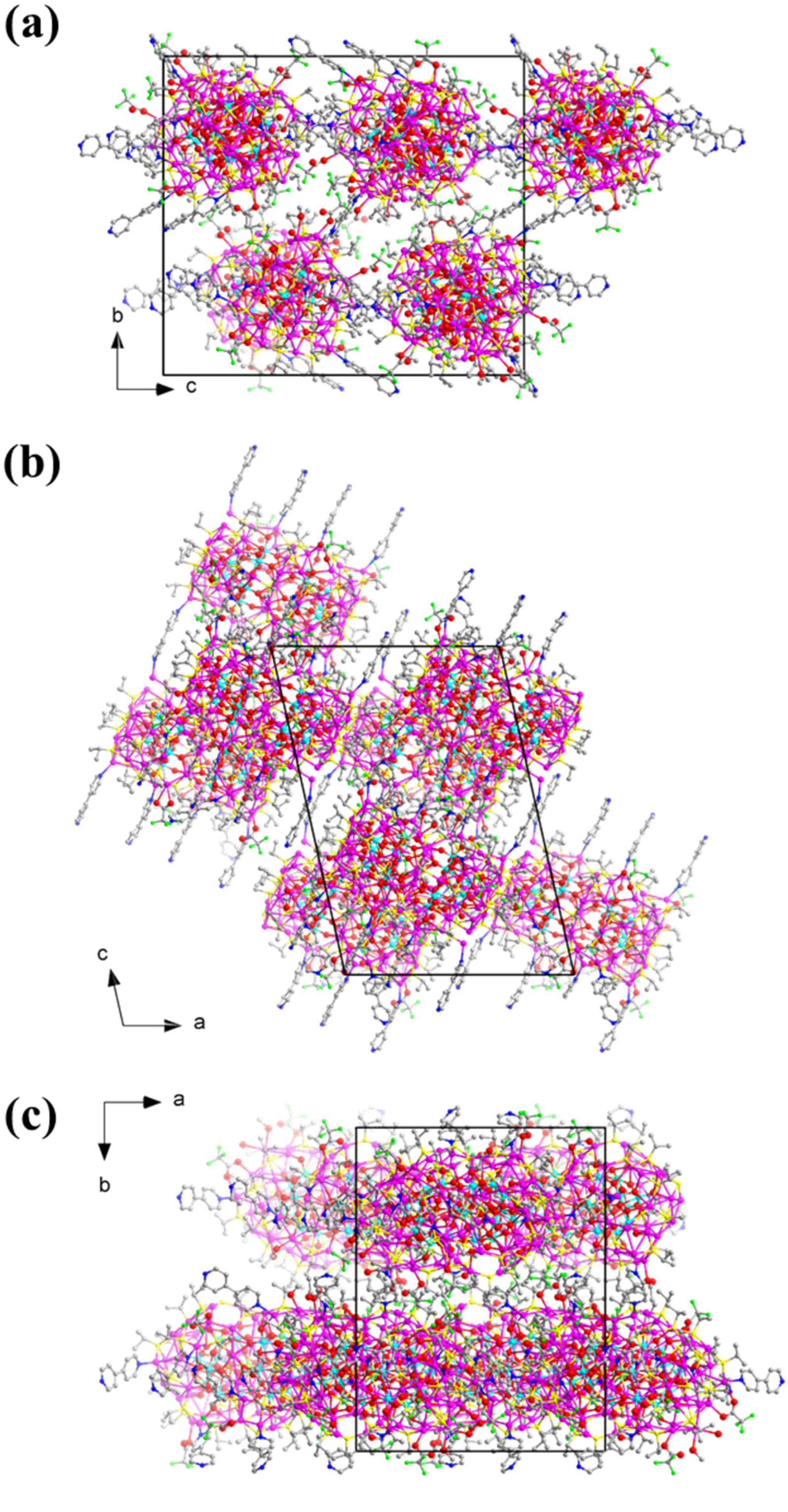

Supplementary Figure 35. Molecules packing diagrams in 1×1×1 unit cell of SD/Ag72a viewed along *a* (a), *b* (b), *c* (c) axis.

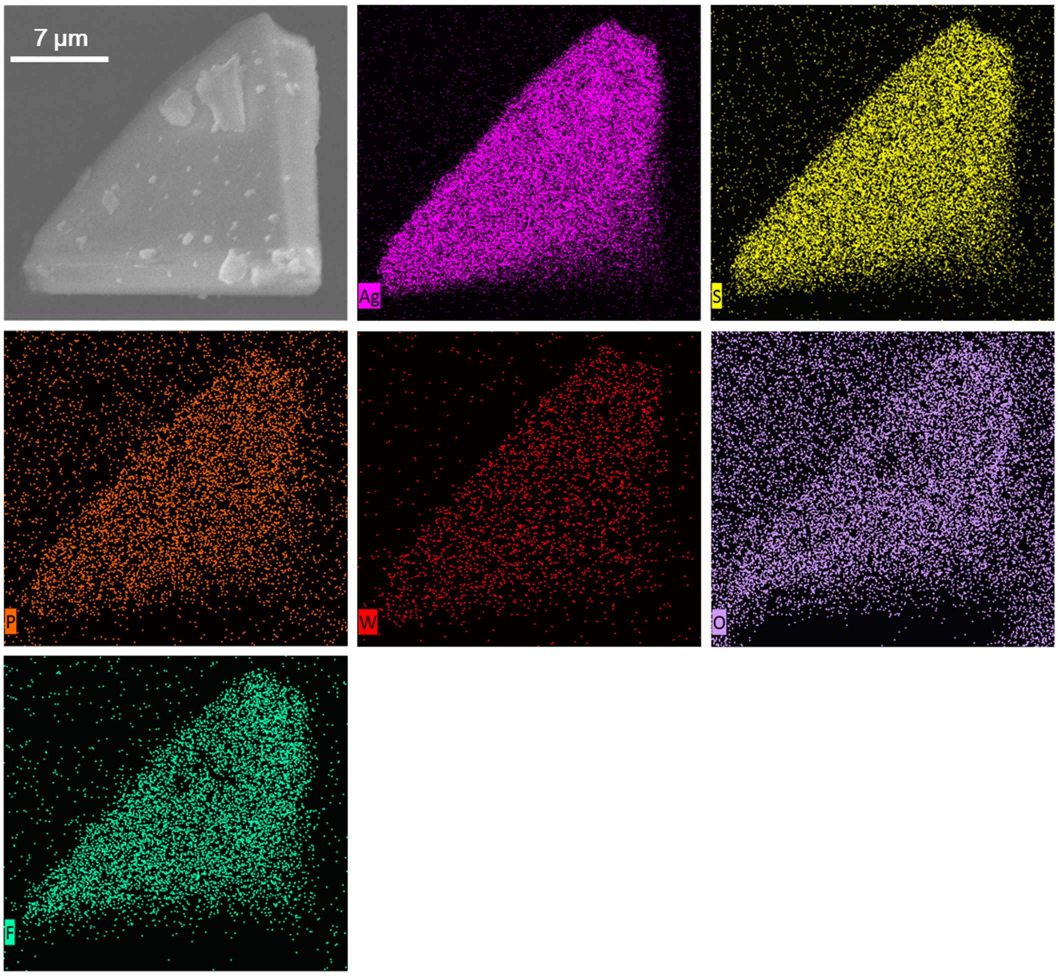

Supplementary Figure 36. SEM and elemental mapping images of SD/Ag51b.

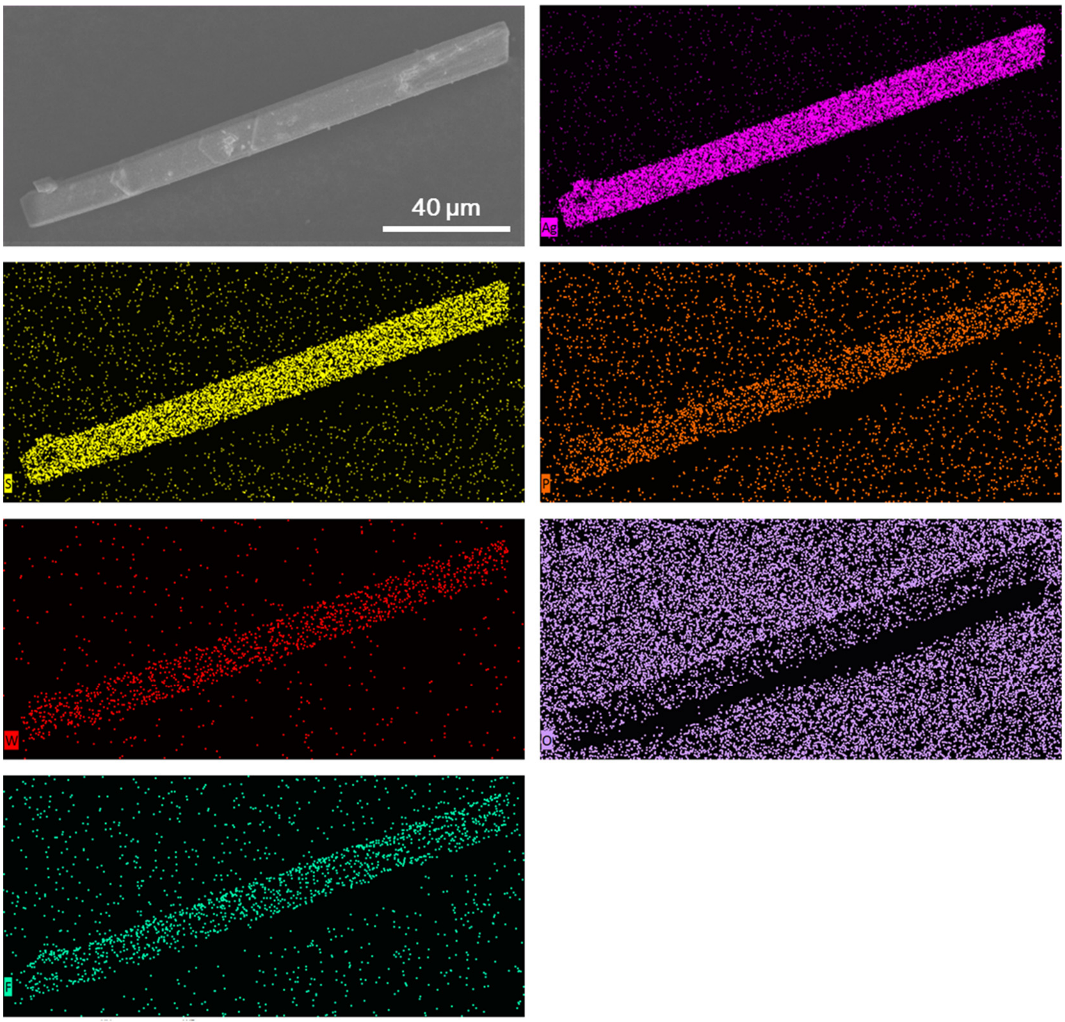

Supplementary Figure 37. SEM and elemental mapping images of SD/Ag72a.

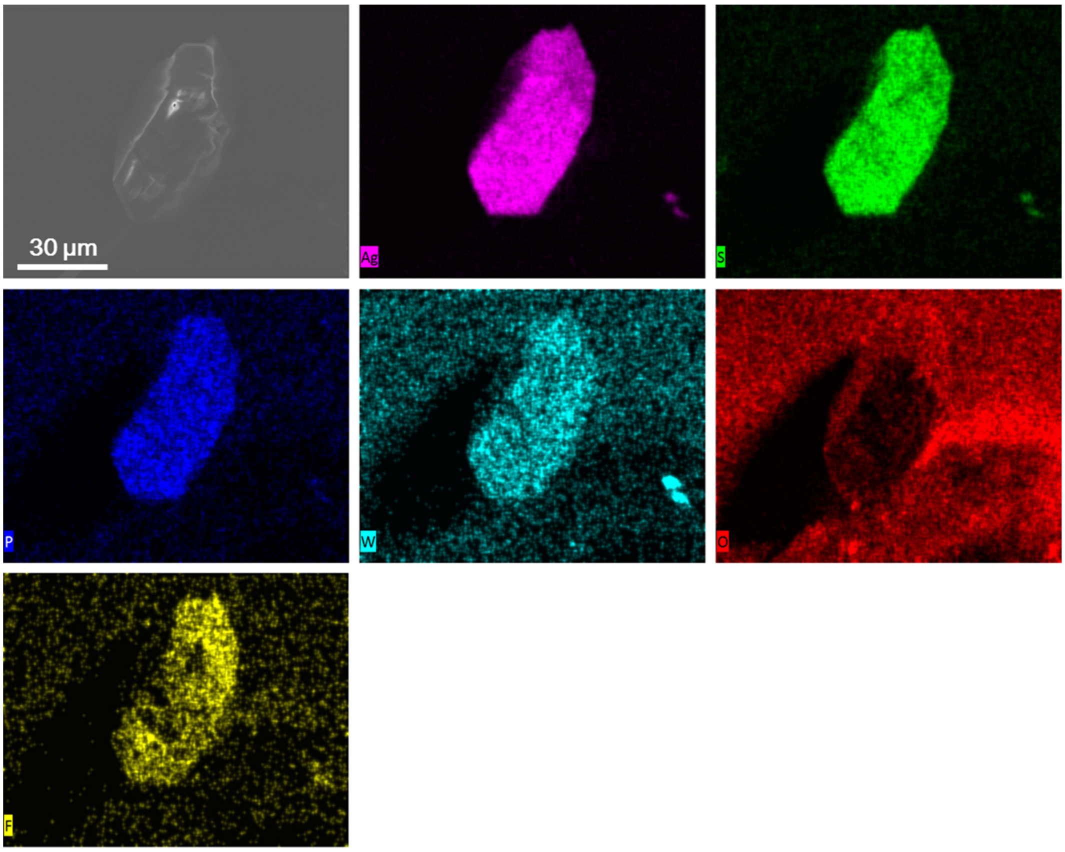

Supplementary Figure 38. SEM and elemental mapping images of SD/Ag72c.

Supplementary Table 1. The formulae of the species detected in ESI-MS of SD/Ag51b dissolved in CH_3OH.

Peak	Species	Exp. *m/z*	Sim. *m/z*
**1a**	$[(PW_9O_{34})@Ag_{49}(i\text{PrS})_{25}(CF_3COO)_{13}]^{2+}$	5431.51	5431.59
**1b**	$[(PW_9O_{34})@Ag_{50}(i\text{PrS})_{25}(CF_3COO)_{14}]^{2+}$	5541.46	5541.53
**1c**	$[(PW_9O_{34})@Ag_{51}(i\text{PrS})_{25}(CF_3COO)_{15}]^{2+}$	5652.40	5652.48
**1d**	$[(PW_9O_{34})@Ag_{52}(i\text{PrS})_{25}(CF_3COO)_{16}]^{2+}$	5762.33	5762.42
**1e**	$[(PW_9O_{34})@Ag_{52}(i\text{PrS})_{25}(CF_3COO)_{16}(H_2O)_3(CH_3OH)_2]^{2+}$	5821.55	5821.47
**1f**	$[(PW_9O_{34})@Ag_{53}(i\text{PrS})_{25}(CF_3COO)_{17}(H_2O)_3(CH_3OH)_2]^{2+}$	5932.52	5932.41
**1g**	$[(PW_9O_{34})@Ag_{54}(i\text{PrS})_{25}(CF_3COO)_{18}(H_2O)_3(CH_3OH)_2]^{2+}$	6042.46	6042.36

Supplementary Table 2. The formulae of the species detected in ESI-MS of adding bipy in the CH_3OH solution of SD/Ag51b.

Peak		Formula	Exp. *m/z*	Sim. *m/z*
**2a**		$[\Delta@Ag_{37}(iPrS)_{21}(CF_3COO)_4(H_2O)_2]^{3+}$	2762.48	2762.46
**2b**		$[\Delta@Ag_{38}(iPrS)_{22}(CF_3COO)_4(H_2O)_2]^{3+}$	2823.13	2823.10
**2c**		$[\Delta@Ag_{39}(iPrS)_{23}(CF_3COO)_4(H_2O)_2]^{3+}$	2884.44	2884.41
**2d**		$[\Delta@Ag_{40}(iPrS)_{23}(CF_3COO)_5]^{3+}$	2945.75	2945.70
**2e**		$[\Delta@Ag_{41}(iPrS)_{24}(CF_3COO)_5]^{3+}$	3007.06	3007.01
**2f**	**2f’**	$[\Delta@Ag_{42}(iPrS)_{25}(CF_3COO)_5]^{3+}$	3067.70	3067.65
	**2f”**	$[\Delta@Ag_{42}(iPrS)_{24}(CF_3COO)_6]^{3+}$	3080.35	3080.31
**2g**	**2g’**	$[\Delta@Ag_{43}(iPrS)_{26}(CF_3COO)_5]^{3+}$	3129.01	3128.96
	**2g”**	$[\Delta@Ag_{43}(iPrS)_{25}(CF_3COO)_6]^{3+}$	3141.67	3141.62
**2h**	**2h’**	$[\Delta@Ag_{44}(iPrS)_{27}(CF_3COO)_5]^{3+}$	3189.65	3189.61
	**2h”**	$[\Delta@Ag_{44}(iPrS)_{26}(CF_3COO)_6]^{3+}$	3202.30	3202.26
**2i**	**2i’**	$[\Delta@Ag_{44}(iPrS)_{23}(CF_3COO)_9(H_2O)_2]^{3+}$	3252.28	3252.23
	**2i”**	$[\Delta@Ag_{44}(iPrS)_{22}(CF_3COO)_{10}(H_2O)_2]^{3+}$	3264.94	3264.88
**2j**	**2j’**	$[\Delta@Ag_{45}(iPrS)_{23}(CF_3COO)_{10}]^{3+}$	3314.25	3314.18
	**2j”**	$[\Delta@Ag_{45}(iPrS)_{22}(CF_3COO)_{11}]^{3+}$	3326.91	3326.84
**2k**	**2k’**	$[\Delta_2@Ag_{60}S(iPrS)_{32}(CF_3COO)_5(bipy)_3(CH_3OH)]^{3+}$	4811.25	4811.33
	**2k”**	$[\Delta_2@Ag_{60}S(iPrS)_{31}(CF_3COO)_6(bipy)_3(CH_3OH)]^{3+}$	4823.90	4823.99
**2l**	**2l’**	$[\Delta_2@Ag_{61}S(iPrS)_{33}(CF_3COO)_5(bipy)_3(CH_3OH)]^{3+}$	4872.26	4872.31
	**2l”**	$[\Delta_2@Ag_{61}S(iPrS)_{32}(CF_3COO)_6(bipy)_3(CH_3OH)]^{3+}$	4884.88	4884.96
**2m**	**2m’**	$[\Delta_2@Ag_{62}S(iPrS)_{34}(CF_3COO)_5(bipy)_3(CH_3OH)]^{3+}$	4933.16	4933.29
	**2m”**	$[\Delta_2@Ag_{62}S(iPrS)_{33}(CF_3COO)_6(bipy)_3(CH_3OH)]^{3+}$	4945.86	4945.94
	**2m”’**	$[\Delta_2@Ag_{62}S(iPrS)_{32}(CF_3COO)_7(bipy)_3(CH_3OH)]^{3+}$	4958.50	4958.59
**2n**	**2n’**	$[\Delta_2@Ag_{63}S(iPrS)_{34}(CF_3COO)_6(bipy)_3(CH_3OH)]^{3+}$	5006.83	5006.92
	**2n”**	$[\Delta_2@Ag_{63}S(iPrS)_{33}(CF_3COO)_7(bipy)_3(CH_3OH)]^{3+}$	5019.48	5019.57
**2o**	**2o’**	$[\Delta_2@Ag_{64}S(iPrS)_{35}(CF_3COO)_6(bipy)_3(CH_3OH)]^{3+}$	5067.83	5067.90
	**2o”**	$[\Delta_2@Ag_{64}S(iPrS)_{34}(CF_3COO)_7(bipy)_3(CH_3OH)]^{3+}$	5080.45	5080.55
**2p**	**2p’**	$[\Delta_2@Ag_{65}S(iPrS)_{36}(CF_3COO)_6(bipy)_3(CH_3OH)]^{3+}$	5128.78	5128.87
	**2p”**	$[\Delta_2@Ag_{65}S(iPrS)_{35}(CF_3COO)_7(bipy)_3(CH_3OH)]^{3+}$	5141.41	5141.53
	**2p”’**	$[\Delta_2@Ag_{65}S(iPrS)_{34}(CF_3COO)_8(bipy)_3(CH_3OH)]^{3+}$	5154.07	5154.18
**2q**		$[\Delta_2@Ag_{66}S(iPrS)_{36}(CF_3COO)_7(bipy)_3(CH_3OH)]^{3+}$	5202.40	5202.50
$\Delta = (PW_9O_{34})^{9-}$				

Supplementary Table 3. The excited states, energy, oscillator strength, and the most probable transitions of model SD/Ag51b from TD-DFT calculations.

State	Energy (nm)	Energy (ev)	Oscillator strength (a.u.)	Most probable transitions	Weight of Transition	Nature of major transitions*
1	474.44	2.6133	0.0010	HOMO→LUMO	0.56	M[C]CT L[C]CT
				HOMO→LUMO+1	0.33	
2	462.67	2.6797	0.0015	HOMO-2→LUMO	0.55	
				HOMO-2→LUMO+1	0.28	
8	437.05	2.8368	0.0035	HOMO-3→LUMO	0.45	
				HOMO-3→LUMO+1	0.19	
15	418.85	2.9601	0.0029	HOMO→LUMO+4	0.16	
				HOMO-5→LUMO	0.12	
				HOMO-6→LUMO+1	0.11	
				HOMO-5→LUMO+1	0.10	
58	379.78	3.2647	0.0042	HOMO-1→LUMO+7	0.21	M[C]CT L[C]CT MMCT LMCT
74	370.14	3.3497	0.0046	HOMO→LUMO+8	0.12	
89	363.93	3.4068	0.0035	HOMO→LUMO+9	0.17	
128	359.82	3.5207	0.0036	HOMO-16→LUMO+2	0.09	
				HOMO-13→LUMO+3	0.07	
				HOMO-10→LUMO+4	0.07	
157	344.35	3.6005	0.0041	HOMO-3→LUMO+10	0.18	
				HOMO-3→LUMO+9	0.10	
161	343.51	3.6093	0.0038	HOMO→LUMO+17	0.09	
				HOMO-3→LUMO+9	0.10	
				HOMO-4→LUMO+8	0.08	
				HOMO-7→LUMO+7	0.08	
168	341.67	3.6288	0.0041	HOMO-4→LUMO+11	0.15	
173	340.33	3.6397	0.0085	HOMO-5→LUMO+8	0.09	
				HOMO-4→LUMO+10	0.09	
183	338.44	3.6634	0.0056	HOMO-6→LUMO+8	0.13	
191	336.85	3.6807	0.0053	HOMO-4→LUMO+9	0.10	
				HOMO-19→LUMO+3	0.10	
236	329.56	3.7621	0.0044	HOMO-6→LUMO+12	0.08	
				HOMO-6→LUMO+9	0.07	
262	326.14	3.8015	0.0045	HOMO-19→LUMO+5	0.09	
				HOMO-19→LUMO+4	0.07	
278	324.05	3.8261	0.0044	HOMO-7→LUMO+12	0.07	
				HOMO-8→LUMO+12	0.07	

* [C] denotes the inner PW_9O_{34} core.

Supplementary Table 4. The crystal data and structure refinements for SD/Ag51b, SD/Ag72a and SD/Ag72c.

Compound	**SD/Ag51b**	**SD/Ag72a**	**SD/Ag72c**
Empirical formula	$C_{121}H_{208}Ag_{51}F_{51}N_3O_{74}PS_{25}W_9$	$C_{195}H_{335}Ag_{72}F_{24}N_{11}O_{86}P_2S_{42}W_{18}$	$C_{213}H_{388}Ag_{72}F_{21}N_{18}O_{83}P_2S_{43}W_{18}$
Formula weight	11846.38	17150.11	17444.85
Temperature/K	100.00(11)	100.00(13)	100.01(11)
Crystal system	triclinic	monoclinic	triclinic
Space group	*P*-1	$P2_1/n$	*P*-1
a/Å	19.5335(3)	29.0993(5)	28.6218(5)
b/Å	21.1951(3)	36.8553(5)	29.0199(5)
c/Å	35.8654(4)	42.7341(6)	30.1817(4)
α/°	93.9933(11)	90	74.6514(14)
β/°	91.0230(11)	102.8059(16)	78.6648(13)
γ/°	117.0168(16)	90	89.7670(14)
Volume/$Å^3$	13175.8(4)	44690.8(13)	23672.8(7)
Z	2	4	2
ρ_{calc} g/cm^3	2.986	2.549	2.447
μ/mm^{-1}	39.428	7.942	33.401
F(000)	10968.0	31612.0	16158.0
Radiation	CuKα (λ = 1.54184)	MoKα (λ = 0.71073)	CuKα (λ = 1.54184)
Reflections collected	120913	225672	256216
Independent reflections	46555 [R_{int} = 0.1261, R_{sigma} = 0.1113]	68026 [R_{int} = 0.1198, R_{sigma} = 0.1453]	83376 [R_{int} = 0.2307, R_{sigma} = 0.1334]
Data/parameters	46555/3071	68026/3562	83376/3877
Goodness-of-fit on F^2	1.477	1.035	1.607
Final R indexes [I>=2σ (I)]	R_1 = 0.1338, wR_2 = 0.3407	R_1 = 0.1064, wR_2 = 0.2298	R_1 = 0.1726, wR_2 = 0.3877
Final R indexes [all data]	R_1 = 0.1499, wR_2 = 0.3693	R_1 = 0.1925, wR_2 = 0.2777	R_1 = 0.2219, wR_2 = 0.4440

Supplementary References

1) Jiang, Z.-G., Shi, K., Lin, Y.-M. & Wang, Q.-M. $[Ag_{70}(PW_9O_{34})_2({}^tBuC{\equiv}C)_{44}(H_2O)_2]^{8+}$: ionothermal synthesis of a silver cluster encapsulating lacunary polyoxometalate ions. *Chem Commun* **50**, 2353-2355 (2014).

2) Yan, B.-J. *et al.* Self-Assembly of a Stable Silver Thiolate Nanocluster Encapsulating a Lacunary Keggin Phosphotungstate Anion. *Inorg Chem* **57**, 4828-4832 (2018).

3) Voss, N. R.; Gerstein, M. 3V: cavity, channel and cleft volume calculator and extractor. *Nucleic Acids Res* **38**, W555-W562 (2010).

4) Dolomanov, O.V., Bourhis, L.J., Gildea, R.J., Howard., J.A.K., Puschmann, H. OLEX2: a complete structure solution, refinement and analysis program. *J Appl Crystallogr* **42**, 339-341 (2009).
