# Supplementary material for: Nuclearity enlargement from [PW9O34@Ag51] to [(PW9O34)2@Ag72] and 2D and 3D network formation driven by bipyridines
Source: Nat Commun. 2022 Apr 4;13:1802. doi: 10.1038/s41467-022-29370-w (PMC8979969; doi:10.1038/s41467-022-29370-w)
Supplement: Supplementary file 2 — Description of Additional Supplementary Files [file 41467_2022_29370_MOESM2_ESM.pdf]

Description of Additional Supplementary Files

Supplementary Data 1: Selected calculated orbitals and compositions (%) of model **SD/Ag51b**

Supplementary Data 2: Selected bond lengths (Å) and angles (°) for **SD/Ag51b, SD/Ag72a** and **SD/Ag72c**
